# Multiregional single-cell transcriptomics reveals an association between partial EMT and immunosuppressive states in oral squamous cell carcinoma

**DOI:** 10.1016/j.isci.2025.112988

**Published:** 2025-06-23

**Authors:** Seunghoon Kim, Hyun Jung Kee, Dahee Kim, Jinho Jang, Hyoung-oh Jeong, Nam Suk Sim, Mischa Selig, Jana Ihlow, Livius Penter, Taejoo Hwang, David Whee-Young Choi, Kyoung Jun Lee, Jaewoong Lee, Young Min Park, Semin Lee, Yoon Woo Koh

**Affiliations:** 1 Department of Biomedical Engineering, College of Information-Bio Convergence Engineering, Ulsan National Institute of Science and Technology (UNIST), UNIST-Gil 50, Eonyang-eup, Ulju-gun, Ulsan 44919, Republic of Korea; 2 Korean Genomics Center, UNIST, UNIST-Gil 50, Eonyang-eup, Ulju-gun, Ulsan 44919, Republic of Korea; 3 Department of Hematology, Oncology, and Tumorimmunology, Campus Virchow Klinikum, Charité–Universitätsmedizin Berlin, 13353 Berlin, Germany; 4 Institute of Pathology, Campus Charité Mitte, Charité–Universitätsmedizin Berlin, 10117 Berlin, Germany; 5 Department of Otorhinolaryngology, College of Medicine, Yonsei University, 50-1 Yonsei-ro, Seodaemun-gu, Seoul 03722, Republic of Korea; 6 Yonsei Institute of Gastroenterology, Department of Internal Medicine, College of Medicine, Yonsei University, 50-1 Yonsei-ro, Seodaemun-gu, Seoul 03722, Republic of Korea; 7 Berlin Institute of Health at Charité – Universitätsmedizin Berlin, Berlin, Germany

**Keywords:** Microenvironment

## Abstract

Oral squamous cell carcinoma (OSCC) is highly heterogeneous and metastatic, and the mechanisms driving OSCC development, progression, and metastasis remain elusive. Here, we performed single-cell RNA sequencing on 231,442 cells obtained from the tumor core (TC), tumor periphery (TP), adjacent surrounding tissue (ST), and metastatic lymph node (mLN) samples of 10 patients with human papillomavirus (HPV)-negative OSCC. TP and TC showed no major immune cell phenotype differences. Interestingly, partial EMT (p-EMT) cells showed significant activation of glycolysis and hypoxia signatures, serving as potential biomarkers for clinical outcomes. Moreover, p-EMT scores of epithelial cells positively correlated with M2 scores of tumor-associated macrophages, while the proportion of p-EMT at TP was negatively associated with that of *GZMB*^+^ exhausted CD8^+^ T cells with cytotoxic potential and *TNFRSF9*^+^ mast cells, conferring an adverse prognosis. Our study provides insights into understanding the interplay between intratumoral heterogeneity and the tumor microenvironment of advanced HPV-negative OSCC.

## Highlights

• CNV similarities in tumor and surrounding tissue suggest field cancerization
• p-EMT at the tumor periphery links to poor prognosis, glycolysis, and hypoxia
• Cytotoxic Tex cells are depleted in high p-EMT tumors and are linked to better outcomes
• p-EMT correlates with M2 macrophages; mast cells link to low p-EMT, better prognosis

## Graphical abstract

**Figure:**
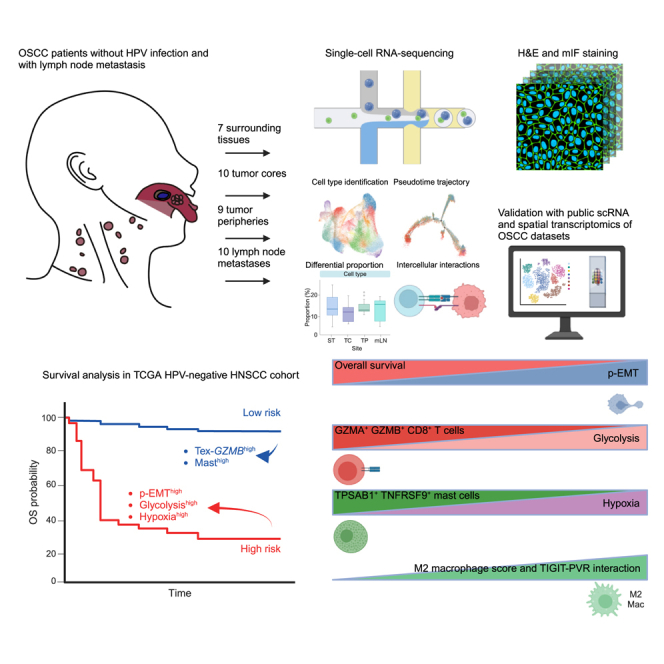

## Introduction

Head and neck squamous cell carcinoma (HNSCC) is the sixth most common human cancer and has generally been correlated with smoking or alcohol consumption.^1^ Each year, approximately 600,000 new HNSCC cases are diagnosed, with an overall mortality rate of approximately 40%, accounting for 3.6% of all cancer-related deaths.^2^ HNSCC develops from various primary sites, of which the oral cavity and lips are the most common and account for 2% of all types of cancers.^3^

Field cancerization caused by smoking or drinking plays an important role in the tumorigenesis of oral squamous cell carcinoma (OSCC). Precancerous lesions accompanied by dysplastic change around the primary tumor are commonly detected.^4^ Moreover, lymph node metastasis plays an important role in determining the treatment method and prognosis of patients with OSCC.^5^ Despite multimodal treatment combining surgery, radiotherapy, and chemotherapy, the survival rate of OSCC has not improved considerably for the past several decades, with the five-year overall survival (OS) being 50%. Therefore, it is essential to study the tumor ecosystem, including the primary tumor, surrounding tissues, and accompanying metastatic lymph nodes (mLNs).

With the advent of single-cell RNA sequencing (scRNA-seq), comprehensive investigations on heterogeneous cellular populations of tumors and their microenvironments have become mainstream.^6^ For the study of HNSCC, several studies have identified immune landscape and subtype-specific signatures associated with human papillomavirus (HPV)-positive and HPV-negative HNSCCs.^7^^,^^8^^,^^9^^,^^10^^,^^11^ Some studies have analyzed cell populations from multi-regions of HNSCCs, focusing on early-stage OSCC,^12^ HPV-related features,^13^ or interactions between malignant cells with fibroblasts and infiltrating T cells.^14^^,^^15^

Especially, a previous study described that the malignant tumor cells expressing the partial epithelial-to-mesenchymal transition (p-EMT) program localize to the edge of the primary tumors with close proximity to cancer-associated fibroblasts (CAF) in oral cavity cancer.^16^ The high expression of p-EMT-related genes in HNSCC has been reported to be associated with unfavorable clinical outcomes and adverse clinical features.^14^^,^^16^ However, the molecular characteristics of p-EMT cells and their association with other cellular populations in the tumor microenvironment (TME), leading to tumor aggressiveness, remain incompletely understood. Our primary goal was to interrogate the associations between spatially distinct intratumoral heterogeneity (ITH) and the TME in advanced HPV-negative OSCC using comprehensive multiregional scRNA-seq on the tumor core (TC), tumor periphery (TP), adjacent surrounding tissue (ST), and mLN samples. As a subgoal, we explored the mechanisms by which p-EMT-related gene signatures contribute to the interplay between ITH and the TME and affect patient prognosis.

## Results

### Multiregional single-cell RNA sequencing of human papillomavirus-negative oral squamous cell carcinoma

**Figure 1:**
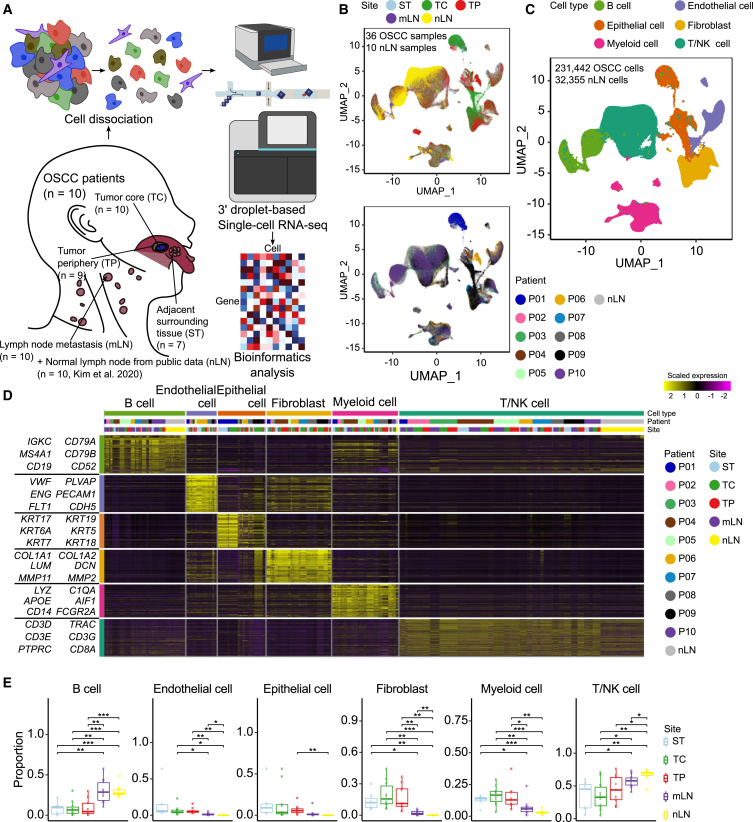
Multiregional scRNA-seq profiling of advanced HPV-negative oral squamous cell carcinoma (A) Schematic representation of the experimental design. The numbers (*n*) of patients and sampling sites are given in the figure. (B) Uniform manifold approximation and projection (UMAP) visualization of sampling site (top) and patient (bottom) information. Each dot represents single cell, colored by sampling site or patient information. (C) UMAP visualization of major cell types. (D) Heatmap of scaled normalized expression of marker genes in the major cell types. Each column represents a cell, and each row represents a marker gene of a major cell type. The top 100 marker genes for each major cell type were used. (E) Proportion distributions of major cell types across sampling sites. Significance of differential proportion (*p* value) between sites was determined by two-sided t-test (box central lines, median; box limits, 25th and 75th percentiles; whiskers, 1.5× the interquartile range; ∗*p* < 0.05, ∗∗*p* < 0.01, ∗∗∗*p* < 0.001).

We collected 36 samples from 10 patients with OSCC (P01-P10) with lymph node metastases (Figure 1A and Table S1). The tissues were collected via surgical resection and classified into TC, TP, ST, and mLN based on their sampling sites with pathologic review (Figure S1). Cells were dissociated, and scRNA-seq was performed using the droplet-based platform (10× Chromium)^17^ (Figure 1A). Overall, more than 450 million reads were sequenced for each sample, with an average of 1,593 median genes and 4,986 median unique molecular identifiers (UMIs) for each cell (Table S2). We also utilized scRNA-seq data from normal lymph nodes (nLN, *n* = 10) of patients with lung cancer^18^ for comparison with those from our mLN samples. After filtering low-quality cells and removing ambient RNA contamination (the details are in the “STAR Methods” section), 231,442 cells were retained and visualized along with 32,355 cells from nLNs using Uniform Manifold Approximation and Projection (UMAP) (Figure 1B). Unsupervised clustering analysis revealed six major cell types consisting of B cells, endothelial cells, epithelial cells, fibroblasts, T/NK cells, and myeloid cells; these findings were also supported by the established marker gene expression and SingleR annotation^19^ (Figures 1C, 1D, and S2A; Table S3). We observed that B and T/NK cells were significantly enriched in the lymph nodes (mLNs and nLNs) compared to those in the TCs and STs, whereas the proportion of stromal and myeloid cells was substantially higher in the primary tumor tissues compared to that in lymph nodes, indicating the heterogeneous composition of the major cell types in different regions (Figures 1E, S2B, and S2C).

To further validate our findings, we compared the cellular composition of publicly available normal tissues (NL) and metastatic tumors in the lymph nodes (LN) from patients with HNSCC^14^ to that of our samples (Figure S2D). We observed a notable difference in major cell type proportions between NL and our primary tumor tissues (including ST, TC, and TP).

Specifically, myeloid cells exhibited a significant increase in primary tissues (mean proportion: 14.9%) compared to NL (mean proportion: 4.91%), representing an approximately 3-fold enrichment (t-test *p* = 1.146e-06). Conversely, fibroblasts were significantly less abundant in primary tissues (mean proportion: 17.2%) compared to NL (mean proportion: 47.5%), showing an approximately 64% reduction (t-test *p* = 0.008737). This inverse relationship is consistent with the known role of fibroblasts in wound healing, leading to their enrichment in normal tissues, as supported by previous research.^20^^,^^21^

Furthermore, the proportion of T cells in our metastatic lymph nodes (mLN, mean: 57.3%, *n* = 10) was approximately 14.5% lower compared to public normal lymph nodes from patients with lung cancer (nLN, mean: 67.1%, *n* = 10) (t-test *p* = 0.031). Similarly, public metastatic lymph nodes (LN, mean: 51.9%, *n* = 4) also showed a reduced T cell proportion compared to the public nLN dataset. While the difference between public LNs and nLNs did not reach statistical significance (*p* = 0.25), likely due to the limited number of public LN samples, these data suggest a trend toward T cell reduction in metastatic lymph nodes.

To understand the functional characteristics of diverse cell populations across sampling sites and their clinical associations, we performed re-clustering analysis of major cell types.

### Tumor cores and peripheries showed distinct epithelial-to-mesenchymal transition signatures

**Figure 2:**
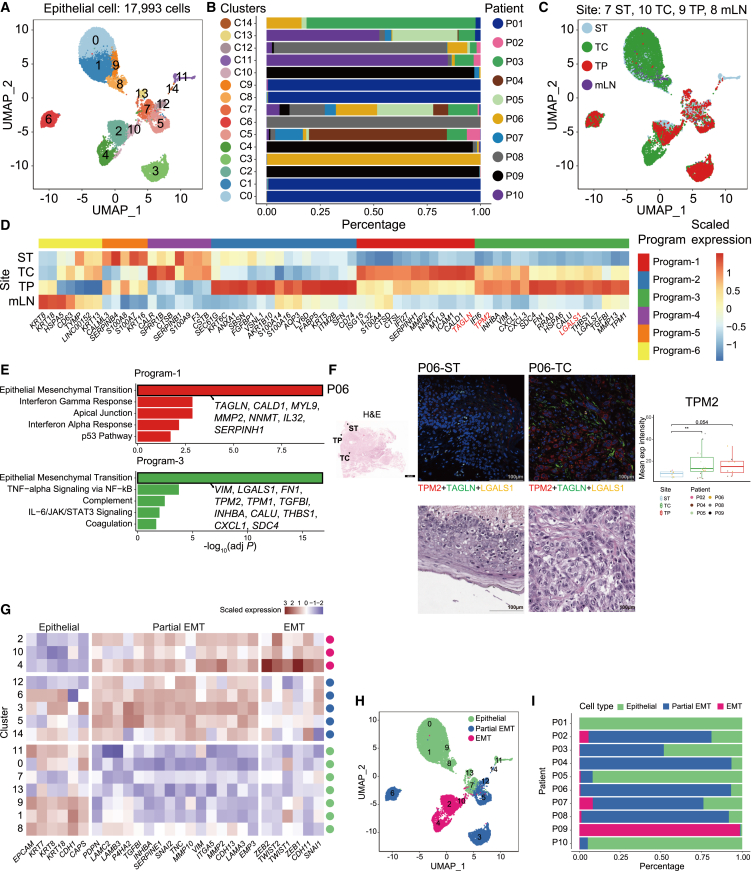
Epithelial cells with EMT characteristics are enriched in tumor cores and peripheries (A) UMAP of epithelial cells derived from all lesions, colored and labeled by cluster. (B) Proportions of patients in each epithelial cell cluster, colored by patient. (C) UMAP of epithelial cells derived from all lesions, colored, and labeled by sampling site. (D) Heatmap of scaled normalized expression of differentially expressed genes (DEGs) across sampling sites. Red color indicates the EMT-associated genes which were used for multiplex immunofluorescence (mIF) staining. (E) Bar plots showing top five significantly enriched pathways in epithelial cells from tumor cores and peripheries (Program-1 and -3). (F) Histological sections of HPV-negative OSCC tumor (patient P06). Left and bottom panels: H&E staining showing the overall tumor morphology (Scale bars: 4 mm and 100 μm). Black arrowheads indicate the sub-site of tumor; ST, TP, and TC. Middle panel: Immunofluorescence staining of EMT markers TPM2 (red), TAGLN (green), LGALS1 (yellow), and nuclear marker DAPI (blue). Co-localization of both TPM2 (red), TAGLN (green) and LGALS1 is observed primarily in the TC region (Scale bar represents 100 μm). Right panel: Boxplot showing the mean expression intensity of TPM2 in different tumor regions. Statistical significance was determined by two-sided t-test (∗*p* < 0.05, ∗∗*p* < 0.01, ∗∗∗*p* < 0.001). (G) Heatmap of scaled normalized expression of epithelial, p-EMT, and EMT-associated genes within epithelial cells. (H) UMAP of epithelial cells derived from all lesions, colored and labeled by epithelial subtype. (I) Proportions of three epithelial subpopulations in patients with advanced OSCC, colored by cell type.

Unsupervised clustering of epithelial cells revealed 15 clusters (Figure 2A). As previously reported in solid tumors,^16^^,^^22^^,^^23^^,^^24^ most epithelial cells showed patient-specific clustering due to the heterogeneous transcriptomic profile, indicating inter-tumoral heterogeneity. While patient-specific clustering was dominant, cells from different sampling sites were often represented within each cluster (Figures 2B, 2C, S3A, and S3B).

Next, we inferred large-scale copy number variations (CNVs) using InferCNV^25^ for deciphering the intra-tumoral heterogeneity across sampling sites. Interestingly, chromosomal aberrations similar to those detected in the primary tumors were observed at dysplastic ST in most patients, albeit to varying degrees (Figure S3C). This observation is also reported in recent studies based on a limited number of samples.^13^^,^^14^ Epithelial cells from mLN showed CNVs consistent with those from primary tissues. For example, samples from P04 and P07 patients showed aberrant CNVs with substantial homogeneity at all sites, including ST. This phenomenon was also observed in most other patients and validated using whole-genome sequencing data (Figure S3C).

To characterize site-specific gene expression signatures, we performed differential gene expression analysis and gene set enrichment analysis (GSEA) across origins (Figures 2D and 2E, the details are described in the supplemental information). Importantly, genes associated with epithelial-to-mesenchymal transition (EMT) were significantly enriched specifically in TC and TP (Figures 2D, 2E, and S3D). To further explore site-specific gene expression programs, we performed hierarchical clustering of genes commonly upregulated in each site across at least three patients. This analysis identified six distinct gene expression programs. EMT-related genes in program1 were highly expressed in TC, as illustrated by *TAGLN* and *MYL9* (Figures 2E and S3D). In program3, the expression of *CXCL1* and *TPM2*, implicated in EMT, was upregulated in the TC and TP (Figures 2E and S3D). We also validated the existence of cells expressing TPM2, TAGLN, and LGALS1 in the TC or TP using hematoxylin and eosin (H&E) and multiplex immunofluorescence (mIF) staining (Figures 2F and S3E).

### Partial epithelial-to-mesenchymal transition is associated with poor prognosis and glycolysis/hypoxia signatures

**Figure 3:**
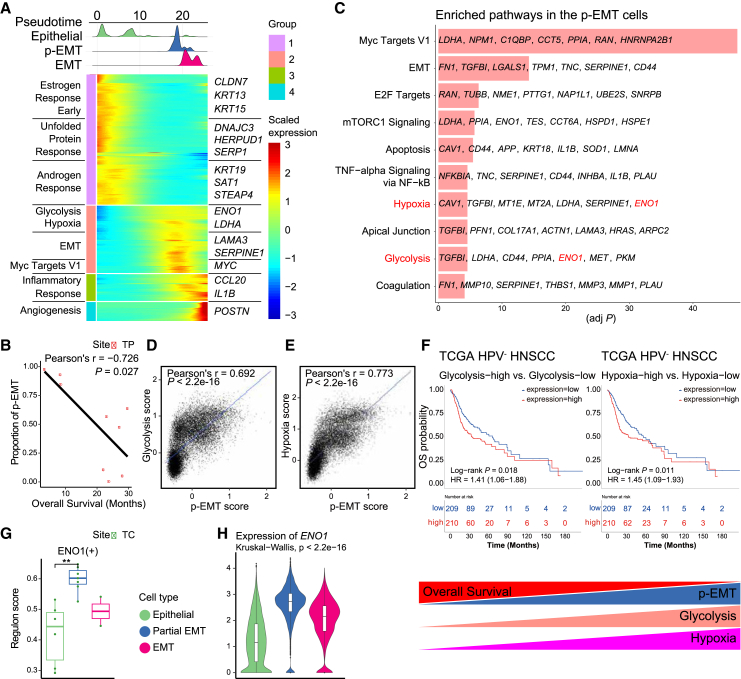
p-EMT cells are linked to poor prognosis through enhanced Glycolysis and hypoxia signatures (A) Transcriptional trajectory analysis of epithelial cells (Top) and the heatmap showing DEGs (Bottom) along the pseudotime trajectory. (B) Scatterplot of OSCC TP samples, showing the negative correlation between the proportions of p-EMT cells and overall survival. Pearson’s correlation (*r*) and associated *p* value are reported inside the scatterplot. (C) Bar plot of enriched pathways in the p-EMT cells compared to other subtypes of epithelial cells. The top 10 significantly enriched pathways (adjusted *p* < 0.05) in the p-EMT cells and the top 7 most highly expressed genes for each term were displayed. Pathways associated with poor prognosis were colored in red. (D and E) Scatterplots of all epithelial cells in our cohort, showing correlation between the expression of p-EMT-related genes with glycolysis-related genes (D), and with hypoxia-related genes (E) at the single-cell level. Pearson’s correlation (*r*) and associated *p* value are reported inside the scatterplot. (F) Kaplan-Meier plots showing that the patients with high expression of glycolysis markers (left) or hypoxia markers (right) have a worse prognosis in the TCGA HPV-negative HNSCC cohort. The high and low groups are divided by the half value of the mean expression of the signature gene sets. (G) Boxplot showing the mean ENO1 regulon activity score in different epithelial subtypes within the tumor core (TC). Each dot represents the mean ENO1 regulon activity score of cells belonging to a specific subtype in each sample. Statistical significance between subtypes was determined using t-tests (∗*p* < 0.05, ∗∗*p* < 0.01, ∗∗∗*p* < 0.001). (H) Violin plots showing the distribution of *ENO1* expression levels in individual cells of each epithelial subtype. Significance of differential expression (*p* value) among subtypes was determined by Kruskal-Wallis test.

Next, we explored three distinct epithelial subtypes based on the established markers of Epithelial, p-EMT, and EMT (Figures 2G and 2H). P01 epithelial cells primarily consisted of clusters representing the Epithelial subtype, whereas the epithelial cells from P09 were primarily enriched in the EMT subtype (Figure 2I). Trajectory analysis reflected the epithelial differentiation process, from Epithelial subtype to p-EMT, p-EMT to EMT, consistent with the recent review on the p-EMT in HNSCC (Figure 3A).^26^ The p-EMT cells, with increased density toward the middle of the pseudotime, showed the enrichment of glycolysis, hypoxia, and EMT pathways (Table S4).

Intriguingly, the abundance of p-EMT cells at TP was negatively correlated with overall survival in our data (Pearson’s *r* = −0.726, *p* = 0.027, Figure 3B), which is consistent with previous studies reporting the association between p-EMT and worse outcome.^14^^,^^27^ We further confirmed that the high proportion of the p-EMT cells was associated with shorter overall survival through a deconvolution analysis of The Cancer Genome Atlas (TCGA) HPV-negative HNSCC cohort (log rank *p* = 0.035, Figure S4A and Table S5). Moreover, our deconvolution analysis revealed a positive correlation between the proportion of epithelial subtype cells and overall survival in the TCGA HNSCC cohort (log rank *p* = 0.035, Figure S4B). Notably, patients with a higher p-EMT/Epithelial cell ratio exhibited a significantly worse overall survival compared to those with a lower ratio (log rank *p* = 0.011, Figure S4B), further emphasizing the prognostic significance of p-EMT.

To elucidate the central mechanism underlying the unfavorable prognosis associated with p-EMT cells, we examined the differential functional states of p-EMT compared to other subtypes. We identified significant upregulation of genes linked to glycolysis and hypoxia within p-EMT cells (Figures 3C, S4C, and S4D). Importantly, we confirmed the positive correlation between the p-EMT scores and the glycolysis and hypoxia scores at the single-cell level in our data (Figures 3D and 3E). We also validated this finding in three publicly available scRNA-seq datasets of HNSCC (Figures S4E and S4F) and in the TCGA HPV-negative HNSCC cohort (Figures S4G and S4H). Therefore, we hypothesized that the enhanced glycolysis and hypoxia signatures within p-EMT cells might serve as a biomarker to predict the unfavorable survival outcomes. Indeed, patients with high glycolysis and hypoxia signatures exhibited significantly shorter overall survival in the TCGA HPV-negative HNSCC cohort (Figure 3F; Table S6). Additionally, our transcription factor activity analysis using SCENIC revealed that the partial EMT subtype is characterized by significantly increased activity of ENO1, a key regulator of glycolysis, and RUNX1, a transcription factor previously implicated in EMT^28^ (Figures 3G, S4I, and S4J). Consistently, the p-EMT subtype exhibited the highest expression of *ENO1* among all subtypes (Figures 3H and S4K). Furthermore, comparative analysis of malignant and non-malignant epithelial cells, stratified by copy number variation, revealed significant upregulation of EMT, hypoxia, and glycolysis-related genes in malignant cells (Figures S4L and S4M), further supporting the role of these pathways in tumor progression and metastasis. These findings collectively demonstrate that p-EMT cells exhibit a distinct metabolic profile and transcriptional landscape, which may contribute to their aggressive behavior and poor prognosis.

### Effector-like Tex cells are depleted as partial epithelial-to-mesenchymal transition cells become abundant

**Figure 4:**
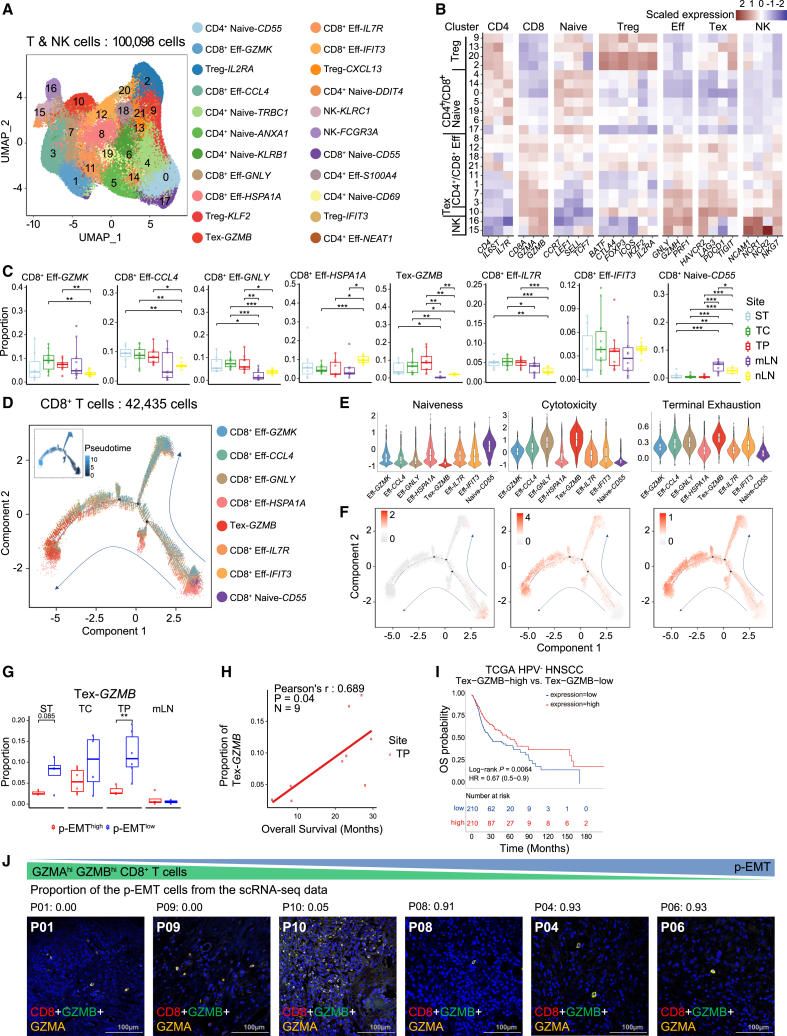
Immunosuppressive characteristics of T/NK cell subsets in advanced HPV-negative OSCC (A) UMAP of T/NK cells derived from all lesions, colored and labeled by cluster number, cell type and marker gene. (B) Heatmap of scaled normalized expression of T/NK cell marker genes. (C) Proportion distributions of CD8^+^ T cell clusters across sampling sites. (D) Developmental trajectories of CD8^+^ T cells by Monocle2 analysis. Individual dots represent single cells, while different colors denote distinct CD8^+^ T cell clusters. The arrows indicate differentiation pathways. The inlet plot showed cells colored by their corresponding pseudotime. (E and F) Violin plots showing T cell naiveness, cytotoxicity, and terminal exhaustion scores for each CD8^+^ T cell cluster (E), along with score changes across pseudotime (F). (G) Proportion distributions of Tex-*GZMB* in p-EMT^high^ and p-EMT^low^ populations across sampling sites. The high and low groups are divided by the half value of the proportion of p-EMT cells. Significance of differential proportion (*p* value) between p-EMT groups was determined by two-sided t-test (box central lines, median; box limits, 25^th^ and 75^th^ percentiles; whiskers, 1.5× the interquartile range; ∗*p* < 0.05, ∗∗*p* < 0.01, ∗∗∗*p* < 0.001). (H) Scatterplot of OSCC TP samples, showing the positive correlation between the proportions of Tex-*GZMB* cells and overall survival. Pearson’s correlation (*r*) and associated *p* value are reported inside the scatterplot. (I) Kaplan-Meier plot showing that patients with HPV-negative HNSCC in the TCGA dataset with high expression of Tex-*GZMB* markers have better prognosis. The high and low groups are divided by the half value of the mean expression of the Tex-*GZMB* markers. (J) Representative immunofluorescence staining for the Tex-*GZMB* markers CD8 (red), GZMB (green) and GZMA (yellow). Scale bar, 100 μm.

Re-clustering of T and NK cells gave rise to 22 clusters consisting of CD4^+^ T, CD8^+^ T, regulatory T (Treg), and NK cells with distinct marker gene expression (Figures 4A and 4B). T and NK cells were broadly distributed regardless of patients and sampling sites (Figures S5A and S5B). We hypothesized that immunosuppressive T cell subsets were prevalent in the primary tumors and metastatic lymph nodes. We found that CD4^+^ naïve-*CD55*, CD4^+^ naïve-*DDIT4* T cells (CD4^+^ naive T cells, CD4^+^ naive), and CD8^+^ naïve-*CD55* T cells (CD8^+^ naive T cells, CD8^+^ naive) were enriched in the mLN compared to those in other sites (Figures 4C and S5C). *CD55*, known for its role in suppressing T cell immunity,^29^ was highly expressed in CD4^+^ naïve-*CD55* and CD8^+^ naïve-*CD55* T cells (Figure S5E). The expression of *DDIT4*, known to inhibit Th17 cell differentiation,^30^ therefore preventing host defense to infection, was upregulated in CD4^+^ naïve-*DDIT4* T cells (Figure S5D).

In contrast, the proportions of CD8^+^ eff-*GNLY* (CD8^+^ effector T cells, CD8^+^ eff), and Tex-*GZMB* T cells (exhausted CD8^+^ T cells, Tex) were significantly increased in the TC and TP compared to those in the lymph nodes (mLN and nLN) (Figure 4C). The lineage structure of CD8^+^ T cells showed that CD8^+^ T cells differentiated from CD8^+^ naive to CD8^+^ eff and from CD8^+^ eff to Tex (Figure 4D). Consistently, velocity analysis further confirmed the differentiation trajectory of CD8^+^ T cells from a naive to effector and subsequently to exhausted phenotype (Figure S5E). Based on the public gene signatures,^31^^,^^32^^,^^33^ we identified CD8^+^ naïve-*CD55* T cells with the highest naiveness and Tex-*GZMB* T cells with the highest cytotoxicity and terminal exhaustion (Figures 4E and 4F). This result indicated that Tex-*GZMB* T cells displayed enhanced effector function.

Treg clusters showed a heterogeneous distribution among sites. Cells from Treg-*IL2RA* and Treg-*IFIT3* primarily existed in TC and TP, whereas cells from Treg-*CXCL13* were slightly enriched in mLN (Figure S5F). Trajectory analysis of Tregs revealed that inhibitory and co-stimulatory characteristics were gradually activated along the pseudotime (Figures S5G–S5I). Especially, Treg-*IL2RA*, which was mainly distributed at the end of pseudotime, had the highest inhibitory, co-stimulatory, and IL2R scores, associated with the inhibition of effector T cells (Figures S5G–S5J). These findings were further supported by analysis using public gene signatures associated with T cell naiveness/central memory, cytotoxicity, and Treg, which revealed consistent results (Figure S5K). Treg-*CXCL13,* located at the beginning of pseudotime, showed high expression of *CXCL13*, encoding a known B cell chemoattractant to lymphoid follicles (Figure S5D).^34^^,^^35^ These findings support that the activated and highly differentiated Tregs were significantly enriched in TC and TP, leading to the immunosuppressive TME of OSCC.

Next, we explored whether there were any associations between T cell subtypes and p-EMT in tumor cells, promoting the immunosuppressive environment. We divided the samples into p-EMT^high^ and p-EMT^low^ groups based on the median value of the p-EMT proportion of all samples. Notably, in TP, patients with low p-EMT proportion had a higher proportion of Tex-*GZMB* T cells compared to those with high p-EMT proportion (Figure 4G). The proportion of Tex-*GZMB* T cells in TP was positively correlated with overall survival in our data (Pearson’s *r* = 0.689, *p* = 0.04, Figure 4H). Furthermore, the expression of Tex-*GZMB* signature genes was associated with better prognosis in the TCGA HPV-negative HNSCC cohort (log rank *p* = 0.0064, Figure 4I). Signature genes from Tex-*GZMB* are known to be involved in T cell cytotoxicity (*GZMA*, *GZMB*, *GNLY*, *PRF1*),^32^ immunity to viral infection (*CCL5*, *KLRC1*),^36^ tissue residency (*CXCL13*),^37^ and T cell exhaustion (*SOX4*, *ENTPD1*)^38^ (Figure S5L). Additionally, mIF staining revealed a potential association of CD8^+^ GZMB^+^ T cells with the p-EMT-low group (Figures 4J and S5M). While not statistically significant (*p* = 0.11 for CD8 and *p* = 0.18 for GZMB), these findings suggest possible transient immune activation in this group.

### Partial epithelial-to-mesenchymal transition is associated with immunosuppressive characteristics of myeloid populations

**Figure 5:**
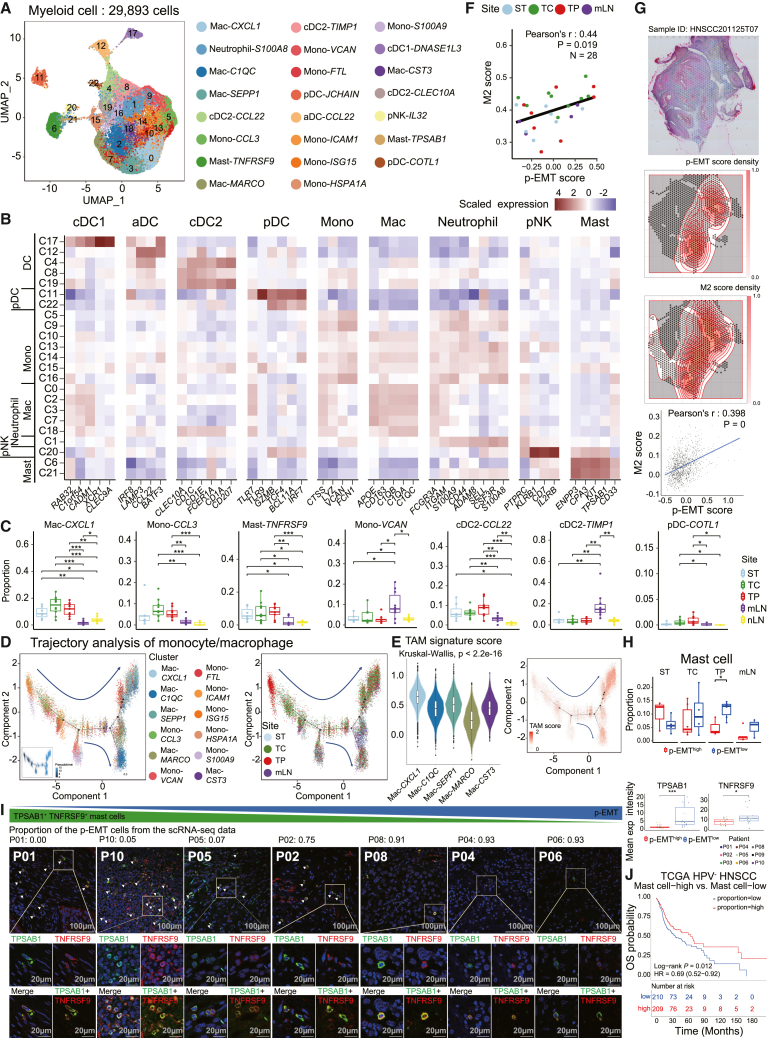
Heterogeneity of myeloid cell subsets in advanced HPV-negative OSCC (A) UMAP of myeloid cells derived from all lesions, colored and labeled by cluster number, cell type, and marker gene. (B) Heatmap of scaled normalized expression of myeloid cell marker genes. (C) Proportion distributions of seven representative myeloid cell clusters with significant proportion differences across sampling sites. (D) Developmental trajectories of monocytes and macrophages. Each dot corresponds to a single cell, colored by cluster label or pseudotime (left), and site (right). (E) Score distributions of tumor-associated macrophage (TAM) signature within each macrophage cluster (left) and along the pseudotime trajectory (right). Significance of differential signature enrichment (*p* value) among clusters was determined by Kruskal-Wallis test. (F) Scatterplot of p-EMT score versus M2 macrophage score across all samples (*n* = 28). Only samples with ≥ 25 macrophage cells and ≥ 25 epithelial cells were included. (G) Histologic section and spatially mapped p-EMT and M2 macrophage scores of one representative stage IV OSCC patient obtained from the publicly available Visium samples.^39^ (H) Proportion distributions of mast cells in p-EMT^high^ and p-EMT^low^ populations across sampling sites. The high and low groups are divided by the half value of the proportion of p-EMT cells. (I) Left panel: Representative immunofluorescence staining of mast cells in tumor tissues. TNFRSF9 (red) and TPSAB1 (green) were used as markers. White arrowheads indicate the cells expressing TNFRSF9 and TPSAB1. Scale bar, 100 μm/20 μm. Right panel: Boxplots showing the mean expression intensity of TPSAB1 and TNFRSF9 in p-EMT^high^ and p-EMT^low^ groups. Statistical significance was determined by two-sided t-test (∗*p* < 0.05, ∗∗*p* < 0.01, ∗∗∗*p* < 0.001). (J) Kaplan-Meier plot of the TCGA HPV-negative HNSCC cohort showing the group with a high proportion of mast cells associated with better prognosis. The high and low groups are divided by the half value of the proportion of mast cells. Significance of differential proportion (*p* value) between sites or p-EMT groups was determined by two-sided t-test (box central lines, median; box limits, 25^th^ and 75^th^ percentiles; whiskers, 1.5× the interquartile range; ∗*p* < 0.05, ∗∗*p* < 0.01, ∗∗∗*p* < 0.001).

Myeloid cells were categorized into 23 clusters and annotated based on the well-defined markers, including *APOE*, *CD163*, and *LYZ* (Figures 5A and 5B). The distribution of cell clusters appeared to be largely concordant among samples after performing batch correction (Figures S6A and S6B). We identified seven dendritic cell (DC) clusters, seven monocyte (Mono) clusters, five macrophage (Mac) clusters, one neutrophil cluster, one NK cell precursor (pNK) cluster, and two mast cell (Mast) clusters. The DC clusters were further classified as activated dendritic cell (aDC), type 1 classical DC (cDC1), type 2 classical DC (cDC2), and plasmacytoid dendritic cell (pDC).

We first focused on the myeloid cell populations that were distinctly enriched in the primary tissues or mLN. Mac-*CXCL1*, Mono-*CCL3*, Mast-*TNFRSF9*, cDC2-*CCL22*, and pDC-*COTL1* showed significantly higher proportions in TC and TP than those in lymph nodes (Figure 5C). Several genes involved in tumor development, progression, and metastasis, including *CXCL1*,^40^ *IL1*,^41^ *CCL3*,^42^ *CCL4*,^43^ *CCL20*,^44^ and *PTGS2*,^45^ were found to be upregulated in Mac-*CXCL1* and Mono-*CCL3* (Figure S6C). Moreover, GSEA revealed that the inflammatory response was significantly activated in Mac-*CXCL1* and Mono-*CCL3* (Figures S6D and S6E). cDC2-*CCL22* showed high expression of *CCL22*, known to promote interaction with Treg^46^ (Figure S6C). pDC-*COTL1* showed expression of *CD5* and *CD81*, similar to the CD5^+^ CD81^+^ pDCs known to induce Treg formation^47^ (Figure S6C).

Conversely, cDC2-*TIMP1* and Mono-*VCAN* were highly enriched in the mLN (Figure 5C). cDC2-*TIMP1* and Mono-*VCAN* showed upregulated gene expression associated with hypoxia and angiogenesis, such as *TIMP1*^48^ and *VCAN*^49^ (Figures S6C, S6E, and S6F). pDC-*JCHAIN* was highly enriched in both mLN and nLN, suggesting its role in antiviral immune response at lymph nodes^50^ (Figure S6G).

The trajectory analysis of monocytes and macrophages identified the differentiation from monocytes to macrophages (Figure 5D). The presence of Mac-*CXCL1* in the upper-right corner with site information suggests that the cells from Mac-*CXCL1* are highly differentiated and enriched in TC and TP. The tumor-associated macrophage (TAM) signature score^51^ was also high in the upper right, indicating that Mac-*CXCL1* is associated with TAMs (Figure 5E).

Next, we hypothesized that immunosuppressive myeloid-cell subsets might be linked to p-EMT cells, given the correlation of p-EMT with unfavorable prognosis. Therefore, we calculated the average M2 macrophage signature score for each sample’s macrophages and measured the average p-EMT signature score for the corresponding sample’s epithelial cells. Strikingly, p-EMT score was positively correlated with M2 score in our data (Pearson’s *r* = 0.44, *p* = 0.019, Figure 5F). This result was further validated using publicly available scRNA-seq datasets of HNSCC (Figure S6H) and the TCGA HPV-negative HNSCC cohort (Figure S6I). We also confirmed that regions with high p-EMT score were adjacent to regions with high M2 score in the published OSCC spatial transcriptomics data (Figures 5G and S6J). To further support this notion, we analyzed publicly available spatial transcriptomics data from OSCC^52^ and found a positive correlation between p-EMT and M2 macrophage signatures in the leading edge and transitory regions (Figure S6K). In contrast, mast cells (consisting of Mast-*TNFRSF9* and Mast-*TPSAB1*) were significantly reduced in the TP of patients with a high p-EMT proportion compared to those with a low p-EMT proportion (Figure 5H). The abundance of the mast cells in patients with a low p-EMT proportion was confirmed by the co-expression of TNFRSF9 and TPSAB1 using mIF staining (Figure 5I). The proportion of mast cells in TP showed trends toward correlation with overall survival in our data (Pearson’s *r* = 0.626, *p* = 0.071, Figure S6L). Deconvolution analysis of the TCGA HPV-negative HNSCC cohort also showed that the proportion of mast cells is positively correlated with OS (log rank *p* = 0.012, Figure 5J).

### Enrichment of highly differentiated plasma and mucosa-associated lymphoid tissue–derived B cells in the primary tumor tissues

We identified 35,523 B and plasma cells consisting of 11 clusters based on the canonical markers such as *CD19* and *CD79A* (Figures S7A and S7B). There were neither patient-specific nor site-specific clusters (Figures S7C and S7D). The 11 clusters were composed of six subtypes, including naive B cell (naive), memory B cell (memory), activated B cell (aBC), germinal center B cell (GC), plasma cell (plasma), and mucosa-associated lymphoid tissue–derived B cell (MALT). naïve-*YBX3*, naïve-*IGLC3*, and GC-*RGS13* were significantly enriched in lymph nodes than in TC and TP (Figure S7E). On the contrary, plasma and MALT clusters showed substantial enrichment in TC and TP (Figure S7E).

Trajectory analysis showed that B cells differentiated from naive into memory, aBC, GC, plasma, and MALT (Figure S7F). Site distribution showed that most cells from mLN were ordered earlier in pseudotime with naive features. In contrast, highly differentiated cells such as plasma and MALT were prevalent in primary tissues later in pseudotime (Figures S7F and S7G). Taken together, these results reveal that the differentiated plasma and MALT B cells were mainly present in the tumor tissues.

### Site-specific enrichment of stromal cells related to inflammation, metastasis, and angiogenesis

Re-clustering of fibroblasts detected five subtypes: CAF, antigen-presenting CAF (apCAF), inflammatory CAF (iCAF), myofibroblast (MF), and smooth muscle cell (SMC; Figures S8A and S8B). Each cluster was composed of multiple patients and sites (Figures S8C and S8D). Recently, iCAF was known to produce inflammatory cytokines^53^ to promote T cell inhibition and metastasis.^54^ In our data, two distinct iCAF clusters were identified (Figure S8B). iCAF-*MFAP5*, enriched in mLN, displayed high expression of *MFAP5*, known to promote tumor growth and invasion potential in solid tumors^55^ (Figures S8E–S8G). In contrast, iCAF-*CRABP1* was abundant in ST and TP (Figure S8E). In the iCAF-*CRABP1* cluster, *CRABP1*, an essential factor for lymph node metastasis in pancreatic neuroendocrine cancer,^56^ and *IGFBP2*, known as a CAF-mediated anoikis inhibitory factor in breast cancer,^57^ were highly expressed (Figures S8F and S8G). We also identified that inflammatory genes, including *CCL2*, *CXCL1*, and *PTGS2*, were highly expressed in iCAF clusters, similar to the metastasis-promoting fibroblast population in lung metastasis of breast cancer^58^ (Figure S8F). In general, CAF and apCAF clusters were enriched in TC or TP (Figure S8E). CAF-*APOE*, which is abundant in primary tissues, exhibited increased expression of *APOE*, known to promote immune suppression in pancreatic cancer^59^ (Figures S8E and S8F). Conversely, apCAF-*CD74* revealed high expression of the genes engaged in immune response, such as *CD74* and *HLA-DRA* (Figure S8F).

Endothelial cells (ECs) consisted of eight clusters and four subtypes: activated postcapillary vein ECs (aPCV), lymphatic ECs (Lymphatic), tip ECs (Tip), and arterial ECs (Arterial; Figures S9A and S9B). Most clusters were made of several patients and sampling sites (Figures S9C and S9D). Tip-*VWA1* and Tip-*ESM1* were enriched in TC than in ST (Figure S9E). These clusters showed the upregulation of genes involved in EMT (*COL4A1*, *COL4A2*), and angiogenesis (*VWA1*), reflecting the endothelial subtypes highly enriched in other malignancies^18^^,^^60^ (Figures S9F and S9G). Contrarily, aPCV-*HLA-DRA* with high expression of genes involved in MHC-II-mediated antigen presentation (*HLA-DPB1*, *HLA-DQA1*, *HLA-DRA*), was depleted in TC, indicating that antigen presentation was downregulated in TC (Figures S9E–S9G). aPCV-*VCAM1* and Tip-*POSTN*, which express genes associated with inflammation (*VCAM1*) and angiogenesis (*POSTN*), respectively, were more prevalent in TP than ST (Figures S9E and S9F).

### Inhibitory cell-cell interactions between partial epithelial-to-mesenchymal transition cells and immune cells

**Figure 6:**
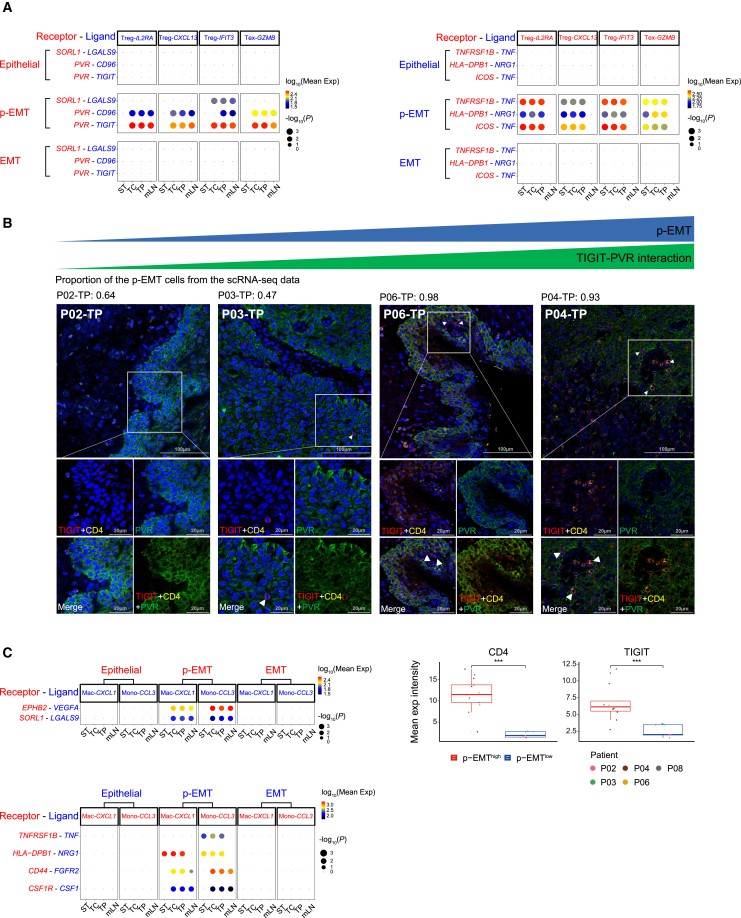
Immunosuppressive interactions between p-EMT cells and immune cells in advanced HPV-negative OSCC (A) Dot plot showing the inferred receptor-ligand interactions between epithelial subtypes and Treg/Tex clusters. The size of each circle represents the significance of interaction (permutation test by CellPhoneDB) and circle color indicates the average receptor and ligand expression level for each pair. (B) Top panel: Representative images of multiplex immunofluorescence staining of TIGIT (red) as a ligand and CD4 (yellow) as a receptor expressing from Treg, and PVR (green) as a cancer cell receptor in OSCC tissue. In patients with a high p-EMT proportion at the TP, TIGIT-expressing Treg cells (in red and yellow) are more frequently juxtaposed with PVR-expressing cells (in green) compared to those with a low p-EMT proportion at TP. Blue shows DAPI staining of nuclei. Scale bar, 100 μm/20 μm. Bottom right panel: Boxplots showing the mean expression intensity of CD4 and TIGIT in p-EMT^high^ and p-EMT^low^ groups. Statistical significance was determined by two-sided t-test (∗*p* < 0.05, ∗∗*p* < 0.01, ∗∗∗*p* < 0.001). (C) Dot plot showing the inferred receptor-ligand interactions between epithelial subtypes and Mono/Mac clusters.

Given that p-EMT cells were enriched in patients with poor prognosis, we hypothesized that p-EMT cells could have stronger inhibitory interactions with immune cells than other epithelial subtypes. We used CellPhoneDB to examine the intercellular communications between epithelial subtypes and Treg/Tex/Mono/Mac. We observed immunomodulatory interactions involving SORL1-LGALS9, TNFRSF1B-TNF, HLA-DPB1-NRG1, and ICOS-TNF exclusively between p-EMT and Treg/Tex (Figure 6A). Notably, PVR-CD96 and PVR-TIGIT interactions associated with T cell inhibition^61^ were observed between the p-EMT and Treg/Tex at TC, TP, and mLN (Figure 6A). Using mIF staining of OSCC patient samples, we identified the CD4^+^ immune cells expressing a higher level of TIGIT juxtaposed to PVR-expressing cancer cells in patients with a high p-EMT proportion compared with those with a low p-EMT proportion (Figure 6B).

We also confirmed the CD44-FGFR2 interaction between Mono/Mac and p-EMT at TC, TP, and mLN, consistent with its known role in promoting gastric cancer growth^62^ (Figure 6C). Similarly, at these sites, we exclusively observed the CSF1R-CSF1 interaction associated with M2-like polarization^63^ between Mono/Mac and p-EMT, along with other interactions, such as angiogenesis (VEGFA-EPHB2) and inhibitory interaction (LGALS9-SORL1).

We termed receptors and ligands (RL) most frequently expressed in p-EMT cells as “RL enriched in p-EMT.” The expression of “RL enriched in *p*-EMT” was positively correlated with p-EMT expression at both the single-cell and TCGA HPV-negative HNSCC bulk RNA-seq levels (Figures S10A–S10C). The p-EMT and the RL enriched in p-EMT markers were associated with poor prognosis (log rank *p* = 0.048, Figure S10D). Collectively, these results represent the presence of an immunosuppressive TME in OSCC, specifically in p-EMT cells.

## Discussion

In this study, we performed single-cell transcriptomic analysis on samples from multiple regions of patients with advanced HPV-negative OSCC to understand the cellular landscape and identify prognostic factors. Overall, we depict a comprehensive landscape of the TME in advanced HPV-negative OSCC, alongside the well-known epithelial subtypes in OSCC. We performed the phenotypic characterization of both epithelial and immune/stromal cells, including the intercellular associations and communications between p-EMT cells and immune cells. Our analysis did not reveal statistically significant differences in the overall proportions of major immune cell populations between TC and TP.

As shown in Figure S3C, our CNV analysis revealed aberrant copy number profiles in STs comparable to those found in primary tumors, suggesting the process of field cancerization in OSCC. This finding is consistent with recent studies reporting somatic copy number alterations in the precancerous lesions^12^^,^^14^^,^^64^ and the benign tissues^13^^,^^65^ of solid tumors. Choi et al. identified CNVs from carcinoma *in situ* cells, mostly from one early-stage HPV-negative patient, showing similar CNVs to those of the malignant cells in the primary tissue of oral cavity cancer.^14^ Puram et al. detected invasive malignant cells in pathologically normal tissue of one early-stage HPV-positive oropharyngeal squamous cell carcinoma.^13^ Sun et al. reported that CNVs were gradually enriched in epithelial cells in oral leukoplakia and cancer regions of early-stage OSCC.^12^ Building on these findings, we uncovered that CNVs similar to those of primary tumors are frequently detected in STs in advanced HPV-negative OSCC.

We observed the upregulation of distinct EMT-related genes in TCs and TPs of malignant epithelial cells. Recent studies have reported the localization of p-EMT program at the leading edge of OSCC with increased invasiveness.^16^^,^^52^ In our study, the p-EMT proportion at TP showed a negative correlation with overall survival. Remarkably, we identified a positive correlation between the expression of p-EMT-associated genes with glycolysis-related and hypoxia-related genes, both at the single-cell level and in bulk RNA-seq data. We further confirmed the association of glycolysis and hypoxia with p-EMT using publicly available single-cell RNA-seq datasets of HNSCC. In addition, we found that the expression of glycolysis and hypoxia-related genes is associated with worse prognosis in the TCGA HPV-negative HNSCC cohort. A recent report linked enhanced glycolysis to increased tumor aggressiveness in cutaneous squamous cell carcinoma.^66^ Another article mentioned that hypoxia activates Twist, a key transcription factor for EMT, in pancreatic cancer.^67^

Tex-*GZMB*, showing the highest cytotoxicity and terminal exhaustion scores, was enriched in patients with a low p-EMT proportion at TP. The proportion of this effector-like Tex at TP showed a positive correlation with OS in our data. The expression of markers in Tex-*GZMB* correlates with favorable prognosis in the TCGA HPV-negative HNSCC cohort, indicating that enhanced cytotoxic activity is associated with better outcomes for patients with HPV-negative HNSCC. This could reflect the previously described T cell subtypes expressing immune checkpoints and effector proteins associated with improved prognosis in solid cancers.^37^^,^^68^^,^^69^ Luoma et al.^37^ reported that CD8 T cells having high activity scores of cytotoxicity and inhibitory signatures from pre-treatment samples correlated with neoadjuvant immune checkpoint blockade response and overall survival in patients with oral cavity cancer and metastatic urothelial cancer. Our finding suggests that the activation of Tex with high cytotoxicity may provide a therapeutic strategy for patients with advanced HPV-negative OSCC.

We also confirmed the presence of heterogeneous Treg populations across the sampling sites of patients with OSCC. The inhibitory score is the highest in Treg-*IL2RA*, enriched in the STs and primary tumors, suggesting the potential of Treg-*IL2RA* as a therapeutic target of advanced HPV-negative OSCC.^70^ This activated Treg subtype was recently reported to be significantly enriched in the tumor tissue of HNSCC compared to the non-malignant inflamed oral mucosa,^71^ indicating field cancerization in ST. Additionally, mIF staining experimentally supported the potential immunomodulatory interaction between cancer cells and CD4^+^ T cells. Therapeutic options to prevent the inhibitory interactions between p-EMT and Treg/Tex/Mono/Mac, including T cell inhibition by PVR expressed by p-EMT cells, could be considered for future work.^61^^,^^72^

The heterogeneous myeloid populations were differentially distributed between primary tissues and lymph nodes. Specifically, inflammatory TAMs (Mac-*CXCL1*) and monocytes (Mono-*CCL3*) were abundant in TCs and TPs than in lymph nodes. Recently, *IL1B*-expressing tissue-resident macrophages have been reported to be co-localized with tumor cells highly expressing EMT markers in renal cell carcinoma, potentially promoting tumor growth.^73^ Consistent with this, we found that many inflammatory cytokines, including *IL1B,* were highly expressed in Mac-CXCL1 and Mono-CCL3. Intriguingly, we identified that the M2 macrophage score is positively correlated with p-EMT score at the single-cell level in our data and publicly available HNSCC single-cell RNA-seq and spatial transcriptomics data. This result was also supported by the TCGA HPV-negative HNSCC bulk RNA-seq data. We also found that mast cells were enriched in the TP of patients with a low proportion of p-EMT and were associated with good prognosis in the TCGA HPV-negative HNSCC cohort. The abundance of mast cells in patients with low p-EMT proportion was validated by mIF staining. This finding is in line with recent reports associating mast cells with better prognosis in HNSCC.^27^^,^^74^

In summary, our single-cell transcriptomic analysis of advanced HPV-negative OSCC revealed several key insights: (1) the presence of comparable copy number alterations at ST and primary tumors, suggesting field cancerization; (2) a negative prognostic impact of p-EMT at TP and its correlation with metabolic pathways; (3) the association of cytotoxic Tex cell enrichment in low p-EMT TP regions with better outcomes; (4) the heterogeneity of Treg populations with an enrichment of the inhibitory Treg subtype in ST and primary tumors; and (5) increased mast cell abundance at TP in low p-EMT cases, linked to favorable prognosis. These findings contribute to a deeper understanding of the TME in advanced OSCC and highlight potential avenues for therapeutic intervention and prognostic stratification.

### Limitations of the study

Our study design did not include non-metastatic head and neck lymph node samples. Although this study was based on a limited number of patient samples, we actively leveraged publicly available HNSCC scRNA-seq, spatial transcriptomics, and bulk RNA-seq datasets to validate our findings. Another limitation is that the interpretation of immune cell proportions in our metastatic lymph node samples is limited by the use of control data derived from normal lymph node samples of patients with lung cancer. While we observed a difference in T cell proportions (as detailed in the results section), this comparison is subject to potential confounding factors arising from the distinct tumor biology of lung versus oral squamous cell carcinoma.

Furthermore, our microscopic analysis of SLUG and ENO1 expression did not reveal a statistically significant difference between patient groups with high and low p-EMT cell ratios (results not shown). This lack of significance may be attributed to the limited number of patient samples and the potential for substantial heterogeneity in the subcellular localization of these proteins. While prior research has demonstrated that ENO1 promotes lung cancer metastasis, at least in part, by upregulating SLUG and inducing EMT,^75^ our current data does not provide statistically significant support for this in our OSCC cohort. Future studies with larger sample sizes and spatial analysis are needed to further investigate this potential link in OSCC.

Functional validation of the underlying mechanism of hypoxia/glycolysis and p-EMT programs and the crosstalk between p-EMT and immune cells in model systems would be helpful to further elucidate the molecular mechanism of how p-EMT contributes to shaping the immunosuppressive environment, which eventually leads to poor prognosis of OSCC.

## Resource availability

### Lead contact

Further information and requests for resources and reagents should be directed to and will be fulfilled by the Lead Contact, Yoon Woo Koh (ywkohent@yuhs.ac).

### Materials availability

This study did not generate new unique reagents.

### Data and code availability

• Processed data for the single-cell RNA-seq experiments and sample information have been deposited at the NCBI GEO (https://www.ncbi.nlm.nih.gov/geo/) under the accession number GSE198315. All single-cell RNA-sequencing data generated by this study have been deposited in the NCBI SRA (https://www.ncbi.nlm.nih.gov/sra) under the accession number PRJNA814536. These accession numbers for the datasets are also listed in the key resources table.
• The source code used for pre-processing and main analysis is available at https://github.com/CompbioLabUnist/SEV_OSCC_scRNA_seq.
• Any additional information required to reanalyze the data reported in this work article is available from the lead contact upon request.

## Acknowledgments

We are grateful to Dr. Jee-Eun Choi, Dr. Bukyung Baik, Prof. Dougu Nam, and Prof. Sung Ho Park for the thoughtful discussion and comments. We also appreciate the patients who generously agreed to provide their samples for this study. This work was supported by the Basic Science Research Program through the 10.13039/501100003725National Research Foundation of Korea, funded by the 10.13039/501100014188Ministry of Science and ICT (NRF-2017R1E1A1A01074804, NRF-2017R1C1B2011227, and NRF-2020R1H1A1009704) and the 10.13039/501100002701Ministry of Education (NRF-2018R1A6A1A03025810). This study is also supported by the faculty research grant of 10.13039/501100008005Yonsei University College of Medicine (6-2015-0057). L.P. is a participant in the BIH Charité Digital Clinician Scientist Program funded by the 10.13039/501100001659DFG, the 10.13039/501100002839Charité—Universitätsmedizin Berlin, and the 10.13039/501100017268Berlin Institute of Health at Charité (BIH) and is supported by the Max-Eder program of the 10.13039/501100005972German Cancer Aid (Deutsche Krebshilfe), by the 10.13039/501100003042Else Kröner-Fresenius-Stiftung (2023_EKEA.102) and the DKMS John Hansen Research Grant. J.I. is a participant in the BIH Charité Clinician Scientist Program funded by the 10.13039/501100001659DFG, the 10.13039/501100002839Charité—Universitätsmedizin Berlin, and the 10.13039/501100017268Berlin Institute of Health at Charité (BIH) and is supported by the 10.13039/501100003042Else Kröner-Fresenius-Stiftung (2023_EKEA.176) and the Berliner Krebsgesellschaft.

## Author contributions

Conceptualization, H.K., and Y.K.; methodology, H.K., H.J., and J.J.; validation, H.K., N.S., S.K., J.J., H.J., T.H., and S.L.; formal analysis, H.K., S.K., J.J., M.S., and J.I; investigation, H.K., S.K., J.J., N.S., and H.J.; resources, H.K., N.S., D.K., Y.P., and Y.K.; data curation, H.K., D.K., S.K., H.J., S.L, and Y.K.; writing—original draft, S.K., N.S., and S.L.; writing—review and editing, S.K., H.K., D.K., J.J., H.J., L.P., T.H., D.W.C., K.L., J.L., Y.P., S.L., and Y.K.; visualization, H.K., S.K., and J.J.; supervision, Y.P., S.L., and Y.K.; funding acquisition, S.L., and Y.K.

## Declaration of interests

The authors declare no competing interests.

## Declaration of generative AI and AI-assisted technologies in the writing process

During the preparation of this work, the authors used Gemini, a large language model, to enhance the clarity and readability of the English language expressions in the scientific writing. After using this tool/service, the author(s) reviewed and edited the content as needed and take(s) full responsibility for the content of the publication.

## STAR★Methods

### Key resources table

**Table:** 

REAGENT or RESOURCE	SOURCE	IDENTIFIER
**Antibodies**		

Anti-α Enolase Antibody (L-27)	Santa cruz	sc-100812; RRID: AB_1118874
CD155 Monoclonal Antibody (D171)	Invitrogen	MA5-13493; RRID: AB_10978147
Human CD4 antibody	R&D	AF-379-NA; RRID: AB_354469
Anti-SLUG antibody	Abcam	ab27568; RRID: AB_777968
TIGIT (E5Y1W) XP® Rabbit mAb	Cell Signaling	#99567; RRID: AB_2922806
TAGLN Monoclonal Antibody (GT336)	Invitrogen	MA5-17276; RRID: AB_2538742
TPM2 Polyclonal Antibody	Bioss	bs-1243R; RRID: AB_10857486
Galectin 1 (LGALS1) Monoclonal Antibody (6C8.4-1)	Invitrogen	43–7400; RRID: AB_2533538
Anti-Mast Cell Tryptase (TPSAB1) antibody [EPR8476]	Abcam	ab134932; RRID: AB_2811029
Anti-CD137 (TNFRSF9) (4-1BB) Monoclonal (2G1)	Invitrogen	MA5-42580; RRID: AB_2911721
CD8a Monoclonal Antibody (AMC908), eFluor 660	eBioscience™	50-0008-82; RRID: AB_2574149
Anti-Granzyme B antibody [EPR22645-206].	Abcam	ab255598; RRID: AB_2860567
Granzyme A Polyclonal Antibody	Bioss	bs-2578R; RRID: AB_10855216
anti-Mouse IgG (H + L) Alexa Fluor™ 488	Invitrogen	A21202; RRID: AB_141607
anti-Mouse IgG (H + L) Alexa Fluor™ 568	Invitrogen	A10037; RRID: AB_11180865
anti-Rabbit IgG (H + L) Alexa Fluor™ 488	Abcam	ab150073; RRID: AB_2636877
anti-Rabbit IgG (H + L) Alexa Fluor™ 568	Invitrogen	A10042; RRID: AB_2534017
anti-Mouse IgG (H + L) Alexa Fluor™ 647	Invitrogen	Ab150107; RRID: AB_2890037
anti-Rabbit IgG (H + L) Alexa Fluor™ 647	abcam	ab150075; RRID: AB_2752244

**Chemicals, peptides, and recombinant proteins**		

Normal Donkey Serum	Jackson ImmunoResearch	017-000-121
Sodium chloride	Sigma-Aldrich	S9888
Sodium phosphate dibasic	Sigma-Aldrich	S9763
Potassium phosphate dibasic	Sigma-Aldrich	P3786
potassium chloride	Sigma-Aldrich	P3911
Antibody Diluent, Ready-to-use diluent, Immunohistochemistry	Dako	S0809
VECTASHIELD® Antifade Mounting Medium with DAPI	Vector Laboratories	H-1200-10

**Deposited data**		

Processed scRNA-seq data	This paper	GEO: GSE198315
Raw scRNA-seq data	This paper	SRA: PRJNA814536
Code	GitHub	https://github.com/CompbioLabUnist/SEV_OSCC_scRNA_seq

**Software and algorithms**		

CellRanger v3.0.2	10x Genomics	https://www.10xgenomics.com/
Scrublet v0.2.1	GitHub	https://github.com/AllonKleinLab/scrublet
SoupX v1.4.5	GitHub	https://github.com/constantAmateur/SoupX
Seurat v3.2.0	GitHub	https://github.com/satijalab/seurat
SingleR v1.0.6	GitHub	https://github.com/dviraran/SingleR
MAST v1.12.0	Bioconductor	https://www.bioconductor.org/packages/MAST
DESeq2 v1.26.0	Bioconductor	https://bioconductor.org/packages/DESeq2
EnrichR v3.0	CRAN	https://cran.r-project.org/web/packages/enrichR/
inferCNV v1.2.1	GitHub	https://github.com/broadinstitute/inferCNV/
Monocle2 v2.14.0	GitHub	https://github.com/cole-trapnell-lab/monocle2-rge-paper
CellPhoneDB v2.1.4	GitHub	https://github.com/Teichlab/cellphonedb
ggplot2 v3.3.2	CRAN	https://cran.r-project.org/web/packages/ggplot2/
pheatmap v1.0.12	CRAN	https://cran.r-project.org/web/packages/pheatmap/
GATK v3.7	McKenna et al.^76^	https://gatk.broadinstitute.org/
BWA v0.7.15	Li and Durbin^77^	https://github.com/lh3/bwa
Samtools v1.6	GitHub	https://github.com/samtools/samtools
Picard v2.9.0	Broad Institute	http://broadinstitute.github.io/picard/
CNVkit v0.9.6	Talevich et al.^78^	https://cnvkit.readthedocs.io/en/stable/
pySCENIC v0.11.0	Van de Sande et al.^79^	https://pyscenic.readthedocs.io/en/latest/index.html
scVelo v0.3.2	Bergen et al.^80^	https://scvelo.readthedocs.io/en/stable/
R project for statistical computing	R Core Team	https://www.r-project.org
Python Programming Language	Python	https://www.python.org

### Experimental model and study participant details

#### Patient recruitment and ethical approval

Samples were obtained from Yonsei Head and Neck Cancer Center. Approvals to collect samples from OSCC patients were granted by the institutional review board at Severance Hospital, Yonsei University College of Medicine (IRB number: 255-001). All patients were given full information of the study and provided written informed consent to participate in the study.

#### Study design

Ten patients diagnosed with advanced oral cavity cancer and treated at Yonsei Head and Neck Cancer Center from March 2019 to February 2020 were recruited in the current study. The inclusion criteria for recruitment were as follows: (1) oral cavity cancer patients older than 18 years with biopsy-proven squamous cell carcinoma with cervical lymph node metastasis on preoperative imaging studies; (2) primary lesion size larger than 3 cm, with or without adjacent dysplastic lesions; and (3) pathologically confirmed negative for HPV infection with p16 staining. All patients were given full information of the study and provided the written consent after approval of the institutional review board at Severance Hospital, Yonsei University College of Medicine (IRB number: 255-001). All patients were treated with complete resection according to the NCCN guideline by experienced head and neck surgeons, Y.W. Koh and Y.M. Park. The general clinical characteristics of the patients are described in Table S1.

### Method details

#### Single-cell library preparation

Fresh tissues from primary tumor lesions (central and periphery of the tumor), dysplastic STs (at least 1 cm apart from the gross margin, within 2 cm distance), and matched mLNs were sampled and retrieved. All tissues were harvested immediately after surgery and dissociated by the gentleMACS Dissociator (Miltenyi Biotec, Bergisch Gladbach, Germany) using Human Dissociation Kit (Miltenyi Biotec, Bergisch Gladbach, Germany) according to the manufacturer’s protocol. The viability of the dissociated cells was confirmed to be over 90% by Cellometer auto C4 (Nexcelom Bioscience, Lawrence, MA, USA) using acridine orange/propidium iodide solution. Single-cell libraries were prepared using the Single Cell 3′ Reagent Kit v3 (10× Genomics, Pleasanton, CA, United States) following the Chromium Single Cell 3′ Reagent Kit v3 protocol (Document # CG000183). Libraries underwent paired-end sequencing on the HiSeq X sequencer (Illumina, San Diego, CA, USA); sequencing was conducted by Macrogen Inc. (Seoul, Korea).

#### Immunofluorescence staining and processing

Formalin-fixed paraffin-embedded tissue sections from the archives of Yonsei Severance hospital tissue bank were collected and prepared on glass slides. For immunofluorescence analysis, the slides were de-paraffinized with xylene and rehydrated. Heat induced antigen retrieval was performed with 90°C for 20 min in Tris-EDTA buffer (10 mM Tris Base, 1 mM EDTA solution, 0.05% Tween 20, pH 9.0). The slides were blocked and stained with primary antibody to SNAI2 (1:200, ab21206, Abcam, Cambridge, UK), ENO1 (1:200, sc100812 Santa Cruz Biotechnology, Dallas, TX, USA), TPM2 (1:100, BS-1243R, Bioss), TAGLN (1:500, MA5-17276, Invitrogen), LGALS1 (1:100, 43-7400, Invitrogen), CD8 (1:100, 50-0008-82, Invitrogen), GZMB (1:500, ab255598, Abcam), GZMA (1:500, BS-2578R, Bioss), TNFRSF9 (1:100, MA5-42580, Invitrogen), TPSAB1 (1:200, ab134932, Abcam), PVR (CD155, 1:20, MA5-13493, Invitrogen), and TIGIT (1:200, #99567, Cell signaling, Danvers, MA, USA). After washing steps in PBS, all slides were stained with secondary antibodies, Alexa-fluor 488-conjugated to Rabbit (1:200, Invitrogen), Alexa-fluor 568-conjugated to mouse (1:200, Invitrogen), Alexa-fluor 647-conjugated to mouse (1:200, Invitrogen). All samples were washed in PBS again and incubated in mounting solution including 4′,6-diamidino-2-phenylindole (DAPI, Vector Laboratories, Burlingame, CA). Images were acquired at 20× and 40× using a Zeiss LSM 700(Carl Zeiss, Oberkochen, Germany) with Zen black software (Carl Zeiss).

H&E and mIF images were obtained from adjacent tissue sections of the same patient, ensuring the accurate correlation of histological features with protein expression patterns.

Microscope images were acquired in .czi format, each containing multiple fluorescent channels corresponding to different proteins of interest. To ensure consistent quality and reduce noise, an automated Fiji macro script was developed. This script systematically processed each image, applying a background reduction algorithm to minimize artifacts and enhance signal clarity for each fluorescent channel. Following background reduction, the script quantified the intensity of individual fluorescent signals for each protein channel.

The fluorescence intensity measurements were aggregated for each condition across all images from individual patients. Aggregated intensity data from multiple images were analyzed using t-tests to compare differences between experimental groups.

#### Processing of scRNA-seq data, integration of public data, and clustering

The Cell Ranger pipeline (version 3.0.2)^17^ was applied for pre-processing, including demultiplexing of sequencing results, barcode processing, read alignment, filtering of estimated droplets, and the generation of gene/barcode matrices. Reads were aligned to the GRCh38 human reference genome (version GRCh38-1.2.0), and the UMI matrix of cell barcodes according to genes was generated for each sample. Cell-free mRNA contamination was estimated and removed using SoupX (version 1.4.5)^81^ with default parameters. Potential doublets were further removed using Scrublet (version 0.2.1)^82^ for each sample, with the expected doublet rate of 0.05. Cells with a doublet score greater than 0.1 were filtered out (approximately 8.5%, 24,933 cells out of the original 293,064 cells). Seurat package (version 3.2.0)^83^ was used for downstream analyses. We excluded low-quality cells using the following criteria: 1) cells with more than or equal to 10% of the transcripts derived from the mitochondrial genome, estimated as apoptotic cells; and 2) cells with a minimum detected gene between 200 and 1,000 based on the QC distribution of each sample. We then normalized the gene expression matrix for each cell by dividing the total expression counts, multiplying a scale factor of 10,000, and converting it to a natural logarithm using the *NormalizeData* function. The *FindVariableFeatures* function was used to detect highly variable genes using the default setting. We measured the cell-cycle scores using the *CellCycleScore* function to regress this signal from the data. We regressed out variations caused by the count of detected transcripts, the mitochondrial gene percent, and the cell-cycle heterogeneity using the *ScaleData* function. We applied the same normalization and doublet filtering method when loading publicly available scRNA-seq dataset of normal lymph node (nLN) samples.^18^ The publicly available nLN samples were composed of T, B, and myeloid cells. Batch effects were corrected in the re-clustering analysis of major cell types, except the epithelial cells, by canonical correlation analysis after normalization using sctransform,^84^ implemented in the Seurat package, to minimize the potential variations from sample processing. We considered each batch as the collection of samples from a single patient. The batch-corrected expression values stored in the “integrated” assay were utilized only for dimension reduction and clustering. After integration, we performed principal component analysis with the top 2,000 variable genes and 50 principal components (PCs) using the *RunPCA* function. For dimension reduction and clustering analysis, the *FindNeighbors* and *FindClusters* functions were used with the number of PCs being 36 and the resolution being 1.0, respectively. Each cell was projected onto a two-dimensional space and visualized using the *RunUMAP* function. This step was iterated over for the re-clustering analysis of major cell types, including epithelial, T, myeloid, B cells, endothelial cells, and fibroblasts (the number of PCs ranging from 13 to 20 and resolution parameters ranging from 0.2 to 1.0 based on the elbow plot and manual review). We excluded metastatic lymph nodes of P03 and P10, which had few epithelial cells, from the re-clustering analysis of epithelial cells. Cell type annotation was conducted with a manual review of established marker genes and SingleR^19^ estimation.

Spatial transcriptomics data reanalyzed to validate the relationship between p-EMT score and M2 macrophage score are available through the Gene Expression Omnibus with accession number GSE181300.^39^ The downloaded counts and H&E-stained tumor

slide images were imported into Seurat (version 4.3.0.1) using *Read10X_Image* and *Load10X_Spatial* functions. After normalizing the expression counts with Seurat’s *NormalizeData* function with default parameters, we calculated the p-EMT and M2 macrophage scores for each Visium spot using the *AddModuleScore* function in Seurat. After selecting the top 5% of spots based on the module scores for each module, we visualized the p-EMT and M2 macrophage module scores using the *geom_density_2d_filled* function in ggplot2.

#### Differential expression and gene set enrichment analysis (GSEA)

Differentially expressed genes (DEGs) that characterize each major cell type were detected using the MAST algorithm^85^ implemented in the *FindAllMarkers* function. To remove false positive genes, we performed pseudobulk differential expression analysis using mean CPM in each sample. We used DESeq2^86^ for the differential gene expression analysis within major cell types for the following criteria:

(1) DEGs within each of the annotated cell types, by comparing each cluster to other clusters of the same cell type.
(2) DEGs for each cluster, by comparing each cluster to all other clusters within the same major cell type.

GSEA was performed using MsigDB_Hallmark_2020 database^87^ implemented in enrichR (version 3.0).^88^ The hierarchical clustering heatmap described in Figure 2D was created using pheatmap (version 1.0.12).

To examine the tissue site specificity of epithelial cells, differential gene expression analysis was performed across sampling sites using MAST for each patient. A set of genes which were upregulated in a specific site in at least three patients were used for GSEA.

To identify the enriched pathways of partial EMT (p-EMT) cells compared to other subtypes, we performed differential expression analysis using the *FindMarkers* function implemented in the Seurat package.

#### Visualization of marker genes on major cell types in scRNA-seq

Marker genes of major cell types were visualized as a heatmap using the *DoHeatmap* function implemented in the Seurat package, as described in Figure 1D. We selected the top 100 marker genes for each major cell type (Table S3) and plotted the scaled expression of each gene across all cells on the heatmap. Marker gene heatmap of each major cell type was visualized using ggplot2 (version 3.3.2).

#### Inference of copy number variation (CNV) via scRNA-seq

Aberrant CNVs in each tumor cell were estimated using the InferCNV package (version 1.2.1).^25^ We performed InferCNV with the default option for each patient, using epithelial cells as a case group and endothelial cells as a control group.

#### Bulk whole-genome sequencing-based CNV analysis

Genomic DNA (gDNA) was extracted from ST, TC, TP, mLN and matched nLN, and sequencing libraries were prepared from 500 ng gDNA using Nextera DNA Flex library prep kit (Illumina, San Diego, CA, USA), according to the manufacturer’s instructions. DNA of ST, TC, TP, and mLN was extracted from some remaining cells after performing scRNA-seq, whereas DNA of nLN was extracted from Formalin-fixed paraffin-embedded tissue. Whole-genome sequencing libraries were sequenced on Illumina NovaSeq6000 with sequencing depth of at least 30× and 150 bp read length. Sequenced reads were mapped onto the human reference genome (version GRCh38) using BWA (version 0.7.15) with the “-M” option.^77^ Mapped bam files were sorted and indexed using Samtools (version 1.6).^89^ Duplicate reads were removed using Picard (version 2.9.0) MarkDuplicates (http://broadinstitute.github.io/picard/). After that, the mapped reads were realigned using GATK (version 3.7)^76^ RealignerTargetCreator and IndelRealigner algorithms. Base quality score was recalibrated using GATK BaseRecalibrator and PrintReads. Somatic CNVs in ST, TC, TP, and mLN were identified by CNVkit (version 0.9.6) with the “—method wgs” and “—target-avg-size 1000000” options using matched normal lymph nodes as controls.^78^

#### Trajectory analysis

We performed single-cell lineage analysis for epithelial, CD8^+^ T, Treg, Mono/Mac, and B cells using the Monocle2 package (version 2.14.0).^90^ The normalized UMI count matrices were imported from Seurat for downstream analysis. We selected the top 50 highly expressed genes for each cluster, as detected by the *FindAllMarkers* function in the Seurat package, to order cells along the pseudotime trajectory using the *orderCells* function with the default parameters. Dimension reduction was performed using the *reduceDimension* function with the DDRTree method. For CD8^+^ T cells, the inferred developmental trajectory was further validated using RNA velocity analysis with scVelo (version 0.3.2).^80^

#### Calculation of functional module scores

To identify the functional characteristics of the clusters of interest, we calculated the gene signature scores of functional modules identified from previous publications for the clusters using the *AddModuleScore* function with the default parameters in the Seurat package on a single-cell level. The genes involved in the functional modules including glycolysis, naiveness, cytotoxicity, terminally exhausted, IL2R, inhibitory, co-stimulatory, Treg, M1, M2, and TAM scores are listed in Table S6.

#### SCENIC analysis

Single-Cell rEgulatory Network Inference and Clustering (SCENIC) analysis was performed using pySCENIC version 0.11.0^79^ to identify transcription factors (TFs) that were differentially activated in the p-EMT subtype. Single-cell RNA-seq data from epithelial cells were used as input for pySCENIC to construct gene regulatory networks. Regulon activity scores were calculated for each cell using the AUCell method. To identify TFs enriched in the p-EMT subtype, we averaged the regulon activity scores of cells within each subtype of each patient sample and performed a two-sided t-test comparing the p-EMT subtype to other epithelial subtypes. Significant TFs were visualized using a heatmap generated with the pheatmap2 package.

#### Cell-cell interaction analysis

We inferred intercellular interactions using CellPhoneDB (version 2.1.4).^91^ The counts per million normalized expression values from all samples were used as the input. The estimated interactions between two cell types were based on the expression profiles of receptors and ligands. We considered the interactions only if at least 25% of cells expressed the specific ligand or receptor and they were between p-EMT cells and immune cells.

#### Survival analysis using bulk RNA-seq

The TCGA HPV-negative HNSCC RNA-seq data were used as a validation set to evaluate the prognostic performance of each gene/cluster signature. TCGA pan-cancer expression data and clinical information were downloaded from National Cancer Institute Genomic Data Commons (https://gdc.cancer.gov) to obtain the information of HNSCC patients. We utilized the sum of scaled expression (Z-normalization) of signature genes to stratify HPV-negative HNSCC patients based on the enrichment of each signature. For the top and the bottom half expression group, the association between signature score and OS was evaluated via Kaplan-Meier analysis and log-rank tests using the survminer (version 0.4.7) and survival (version 3.2.3) R packages. Additionally, we used BayesPrism (version 1.4.0)^27^ to conduct the deconvolution analysis of major cell type or subtype composition in the TCGA HPV-negative HNSCC bulk RNA-seq cohort. In our dataset, cells in all sites were used to infer the composition of major cell types and subtypes. The deconvolution result of subtypes is described in Table S5. We performed survival analysis using the median of the proportion of major cell type or subtype composition to compare top and bottom half.

### Quantification and statistical analysis

All statistical tests were performed in R (version 3.6.3). Pair-wise t-tests were performed to analyze the proportional differences between sites within each major cell type/cluster. Correlations between two signature scores were calculated using Pearson correlation. Kruskal-Wallis tests were performed to compare signature scores among multiple groups. A log-rank test was performed for all survival analyses to identify significant differences between the high signature expression group (top 50%) and the low signature expression group (bottom 50%). No mathematical corrections were made for multiple comparisons. Significance is displayed as ∗, P < 0.05; ∗∗, P < 0.01; and ∗∗∗, P < 0.001.

## Supplemental information

Document S1. Figures S1–S10

Table S1. Clinical information of patients with oral squamous cell carcinoma in this study

Table S2. Single-cell RNA-sequencing statistics of the oral squamous cell carcinoma samples included in this study

Table S3. Top 100 marker genes of major cell types

Table S4. Pathway enrichment results of trajectory analysis of epithelial cells

Table S5. Deconvolution result of the TCGA HPV-negative HNSCC cohort using subtype information from all sites

Table S6. List of gene signatures for functional modules
