# Supplementary material for: Multiregional single-cell transcriptomics reveals an association between partial EMT and immunosuppressive states in oral squamous cell carcinoma
Source: iScience. 2025 Jun 23;28(9):112988. doi: 10.1016/j.isci.2025.112988 (PMC12496189; doi:10.1016/j.isci.2025.112988)
Supplement: Document S1. Figures S1–S10 [file mmc1.pdf]

iScience, Volume *28*

Supplemental information

Multiregional single-cell transcriptomics reveals an association between partial EMT and immunosuppressive states in oral squamous cell carcinoma

Seunghoon Kim, Hyun Jung Kee, Dahee Kim, Jinho Jang, Hyoung-oh Jeong, Nam Suk Sim, Mischa Selig, Jana Ihlow, Livius Penter, Taejoo Hwang, David Whee-Young Choi, Kyoung Jun Lee, Jaewoong Lee, Young Min Park, Semin Lee, and Yoon Woo Koh

Supplemental Information

Multiregional single-cell transcriptomics reveals an association between partial EMT and immunosuppressive states in oral squamous cell carcinoma

Seunghoon Kim, Hyun Jung Kee, Dahee Kim, Jinho Jang, Hyoung-oh Jeong, Nam Suk Sim, Mischa Selig, Jana Ihlow, Livius Penter, Taejoo Hwang, David Whee-Young Choi, Kyoung Jun Lee, Jaewoong Lee, Young Min Park, Semin Lee, and Yoon Woo Koh

Document S1. Figures S1–S10.

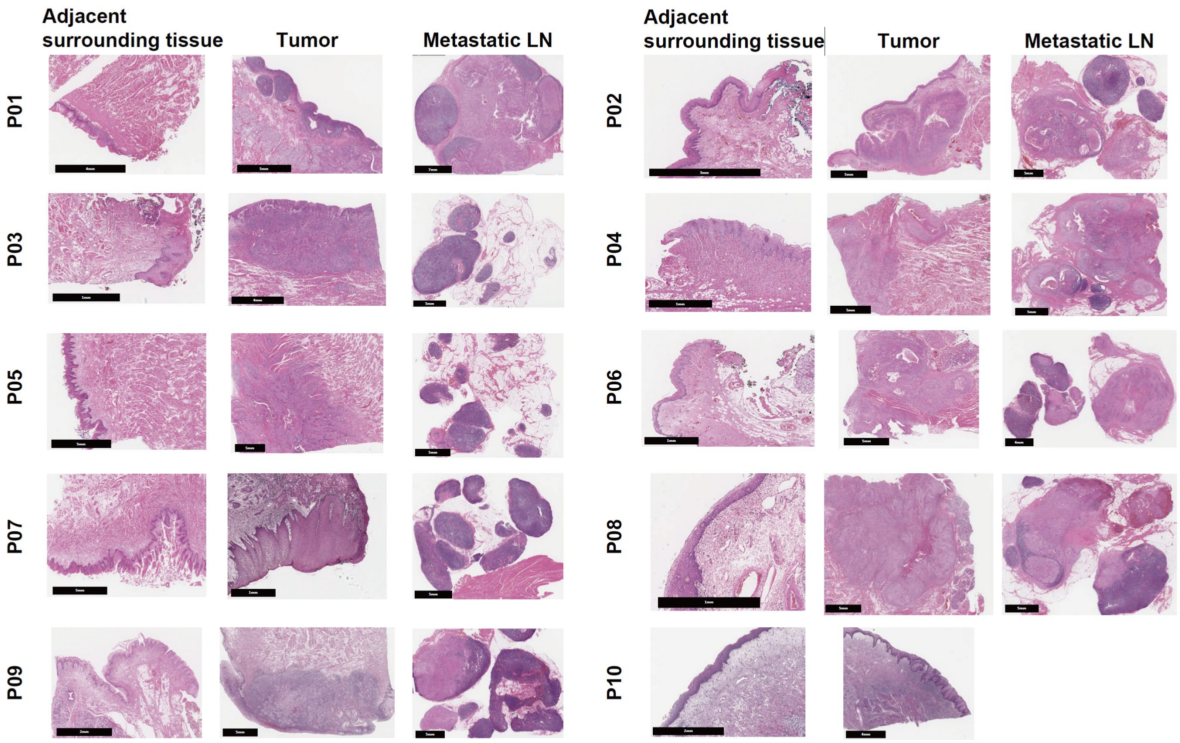

Figure S1. Hematoxylin and eosin (H&E) staining images of the adjacent surrounding tissue, tumor tissue, and metastatic lymph node in 10 OSCC patients included in the study.

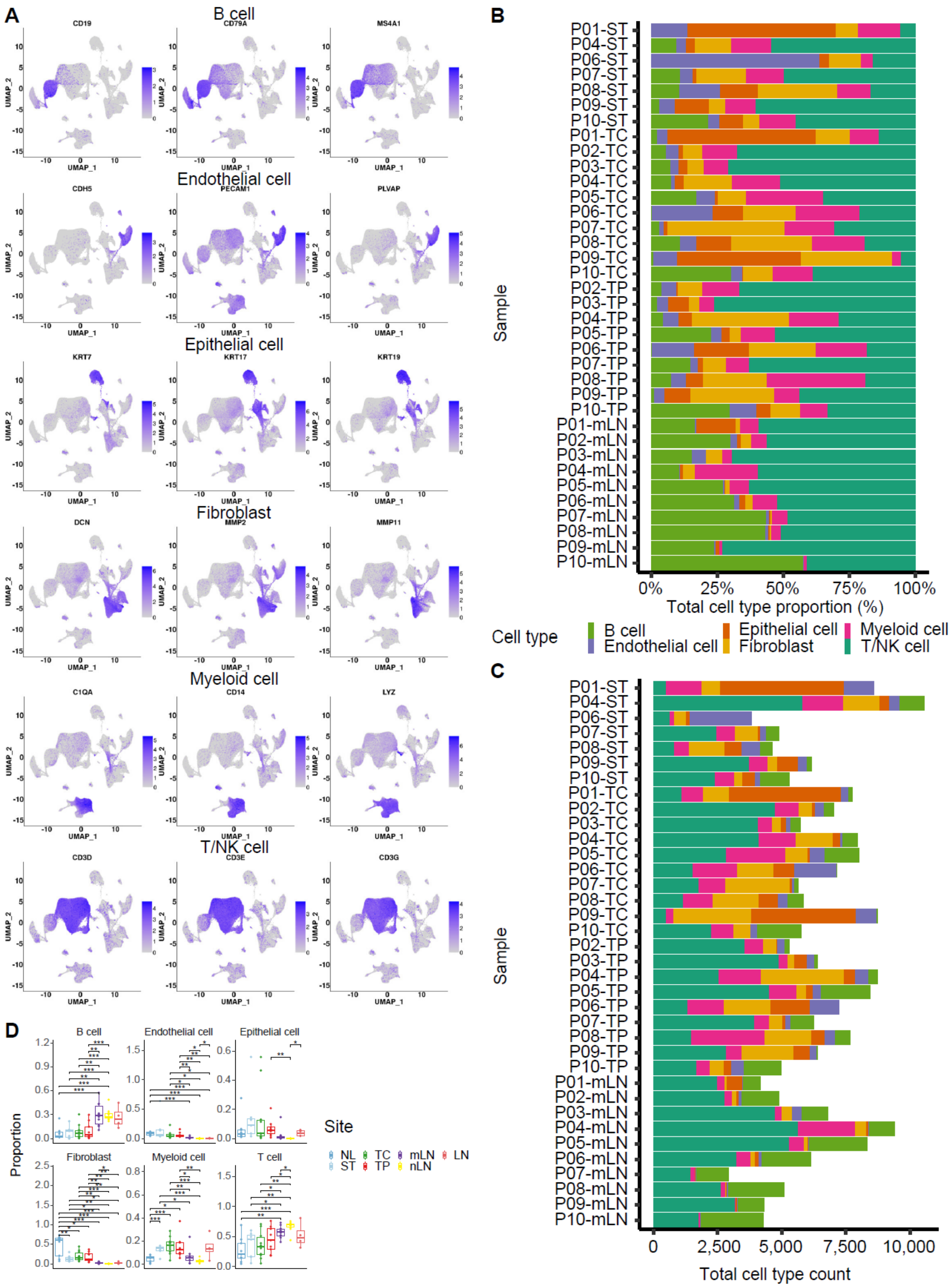

Figure S2. Characterization of major cell types in advanced HPV-negative OSCC.

(A) UMAP plots showing the expression of marker genes for the major cell types.

(B) Proportions of the major cell types in 36 OSCC samples, colored by major cell type.

(C) Counts of the major cell types in 36 OSCC samples, colored by major cell type.

(D) Box plots showing the distribution of major cell type proportions in our samples (from diverse sites) and public HNSCC patient samples: normal tissue (NL) and metastatic tumors in the lymph nodes (LN). Significance of differential proportion (*P* value) between sites was determined by two-sided t-test (box central lines, median; box limits, 25th and 75th percentiles; whiskers, 1.5x the interquartile range; * $p < 0.05$, ** $p < 0.01$, *** $p < 0.001$).

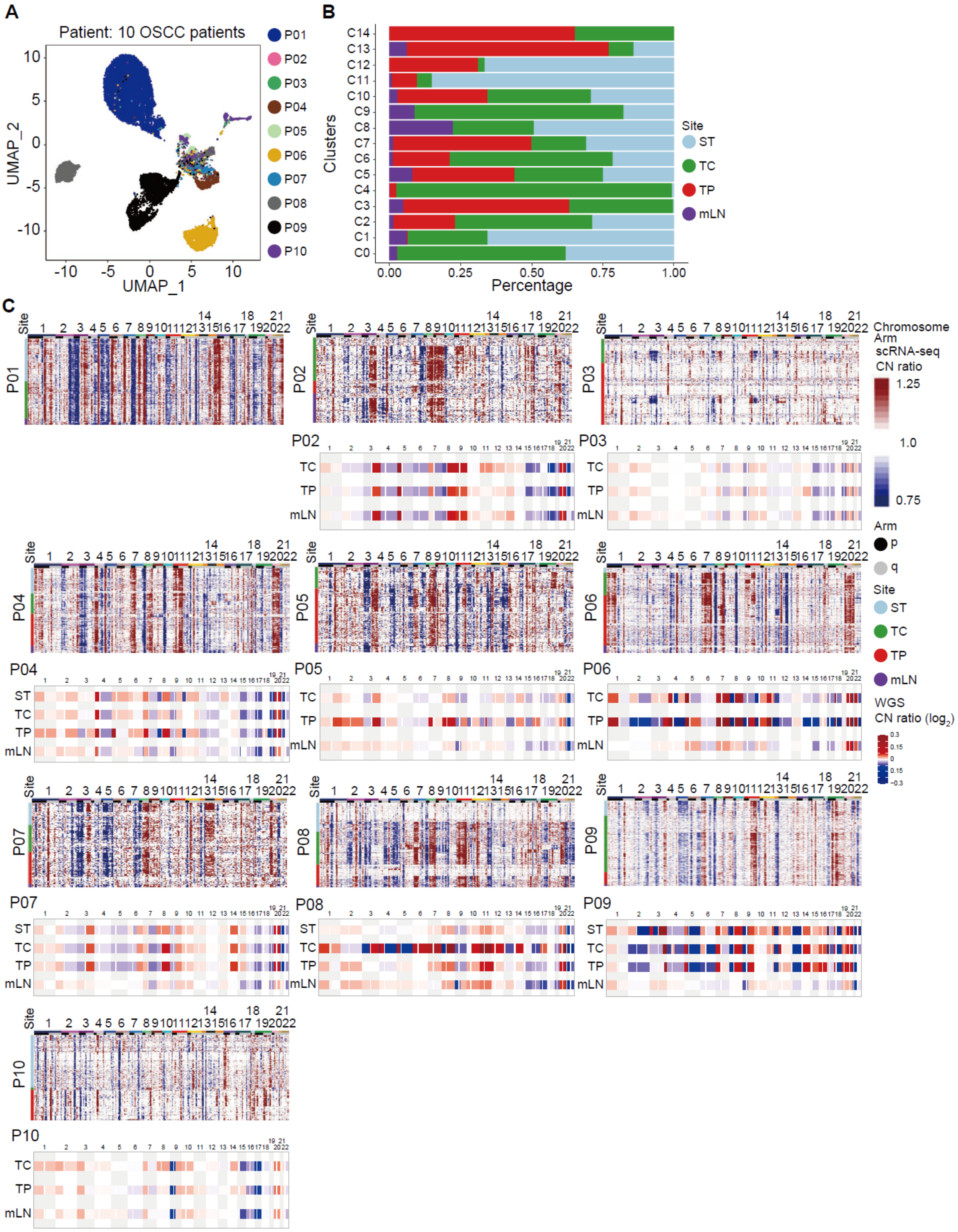
A
Patient: 10 OSCC patients
UMAP_1
UMAP_2
P01
P02
P03
P04
P05
P06
P07
P08
P09
P10
B
Clusters
Percentage
Site
ST
TC
TP
mLN
C
Chromosome
Arm
scRNA-seq
CN ratio
1.25
1.0
0.75
Arm
p
q
Site
ST
TC
TP
mLN
WGS
CN ratio (log2)

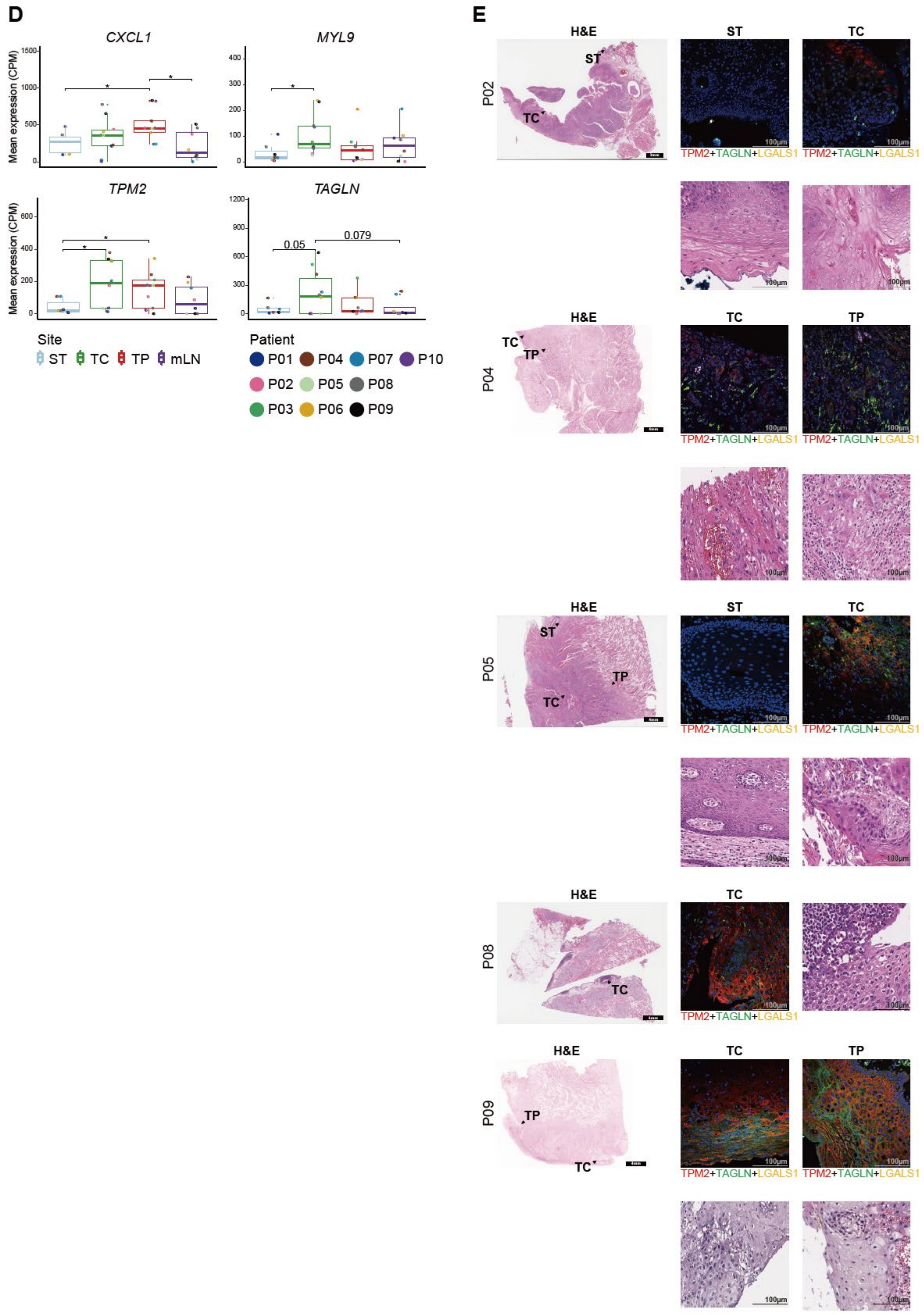

Figure S3. Re-clustering of epithelial cells.

(A) UMAP of epithelial cells derived from all lesions, colored and labeled by patient.

(B) Proportions of sites in each epithelial cell cluster, colored by site.

(C) Heatmaps showing large-scale CNVs for all epithelial cells (row) from 10 OSCC patients, inferred from scRNA-seq (top) and WGS (bottom). Column at the left of the heatmap from scRNA-seq data represents the origin of cells.; CN: copy number, Red: amplification, Blue: deletion.

(D) Representative EMT-related genes upregulated in TC and TP. Each dot represents the mean expression of a given gene in the epithelial cells from each sample. Significance of differential expression (p value) between sites was determined by two-sided t-test (box central lines, median; box limits, 25th and 75th percentiles; whiskers, 1.5x the interquartile range; * $p < 0.05$, ** $p < 0.01$, *** $p < 0.001$).

(E) Five histologic sections representing advanced HPV-negative OSCC tumors (P02, P04, P05, P08, P09), stained by H&E (left) (Scale bars represent 3 mm, 4 mm, 4 mm, 4 mm, 4 mm, respectively) and confocal images showing immunofluorescence staining (right) for EMT markers TPM2 (red), TAGLN (green), LGALS1 (yellow), and nuclear marker DAPI (blue). H&E images were magnified to a scale of 100 μm and positioned below or to the right of the corresponding confocal images for direct comparison. Black arrowheads in H&E indicate the sub-site of tumor; TC, TP and ST. Co-localization of both TPM2 (red), TAGLN (green) and LGALS1 is noted in the TC and TP of tumor, whereas it is absent in ST (Scale bar represents 100 μm).

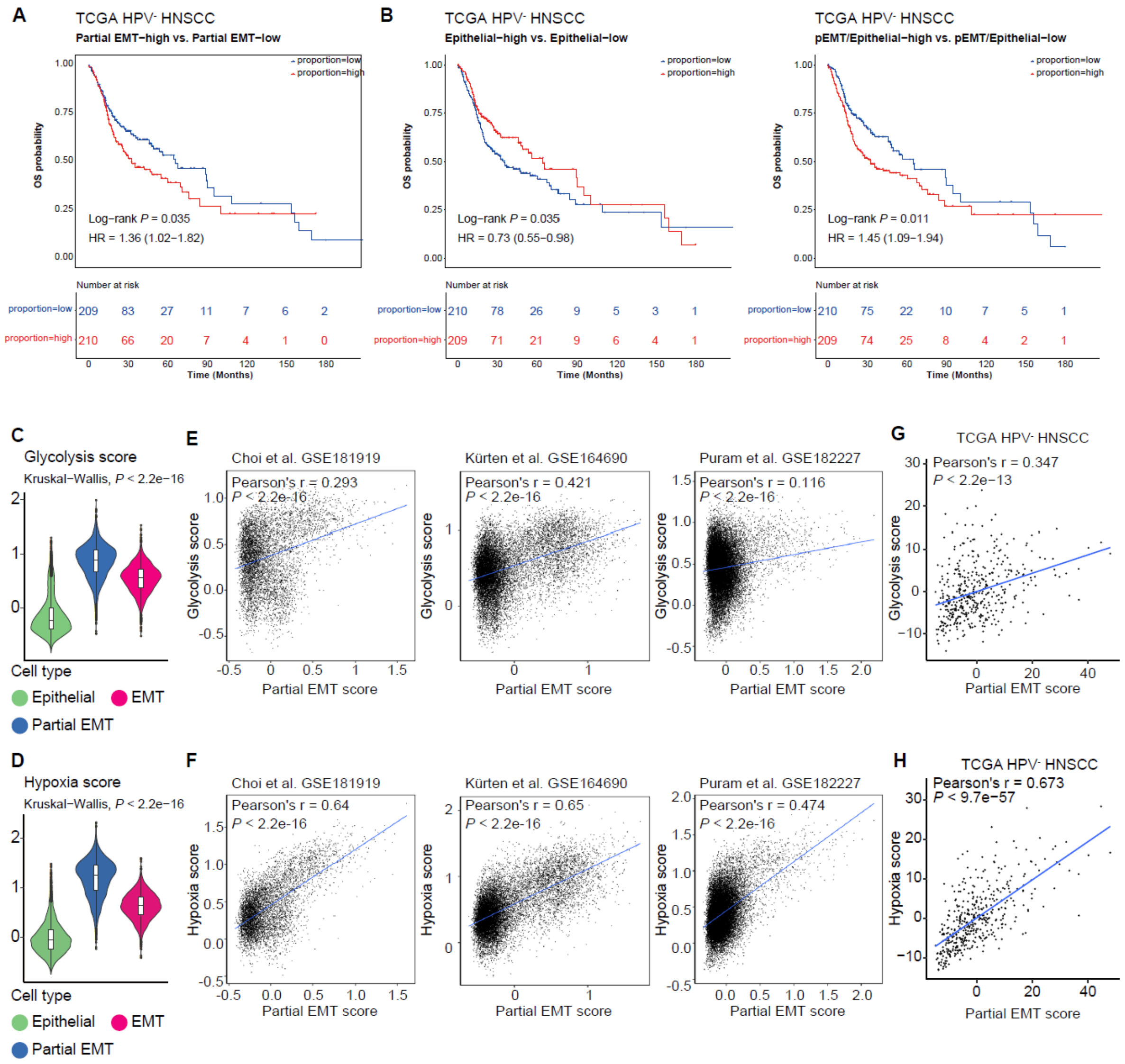
A
TCGA HPV- HNSCC
Partial EMT−high vs. Partial EMT−low
proportion=low
proportion=high
OS probability
Log−rank P = 0.035
HR = 1.36 (1.02−1.82)
Number at risk
proportion=low 209 83 27 11 7 6 2
proportion=high 210 66 20 7 4 1 0
Time (Months)
B
TCGA HPV- HNSCC
Epithelial−high vs. Epithelial−low
Log−rank P = 0.035
HR = 0.73 (0.55−0.98)
proportion=low 210 78 26 9 5 3 1
proportion=high 209 71 21 9 6 4 1
TCGA HPV- HNSCC
pEMT/Epithelial−high vs. pEMT/Epithelial−low
Log−rank P = 0.011
HR = 1.45 (1.09−1.94)
proportion=low 210 75 22 10 7 5 1
proportion=high 209 74 25 8 4 2 1
C
Glycolysis score
Kruskal−Wallis, P < 2.2e−16
Cell type
Epithelial
EMT
Partial EMT
E
Choi et al. GSE181919
Pearson's r = 0.293
P < 2.2e-16
Kürten et al. GSE164690
Pearson's r = 0.421
P < 2.2e-16
Puram et al. GSE182227
Pearson's r = 0.116
P < 2.2e-16
Glycolysis score
Partial EMT score
G
TCGA HPV- HNSCC
Pearson's r = 0.347
P < 2.2e−13
D
Hypoxia score
Kruskal−Wallis, P < 2.2e−16
F
Choi et al. GSE181919
Pearson's r = 0.64
P < 2.2e-16
Kürten et al. GSE164690
Pearson's r = 0.65
P < 2.2e-16
Puram et al. GSE182227
Pearson's r = 0.474
P < 2.2e-16
Hypoxia score
H
TCGA HPV- HNSCC
Pearson's r = 0.673
P < 9.7e−57

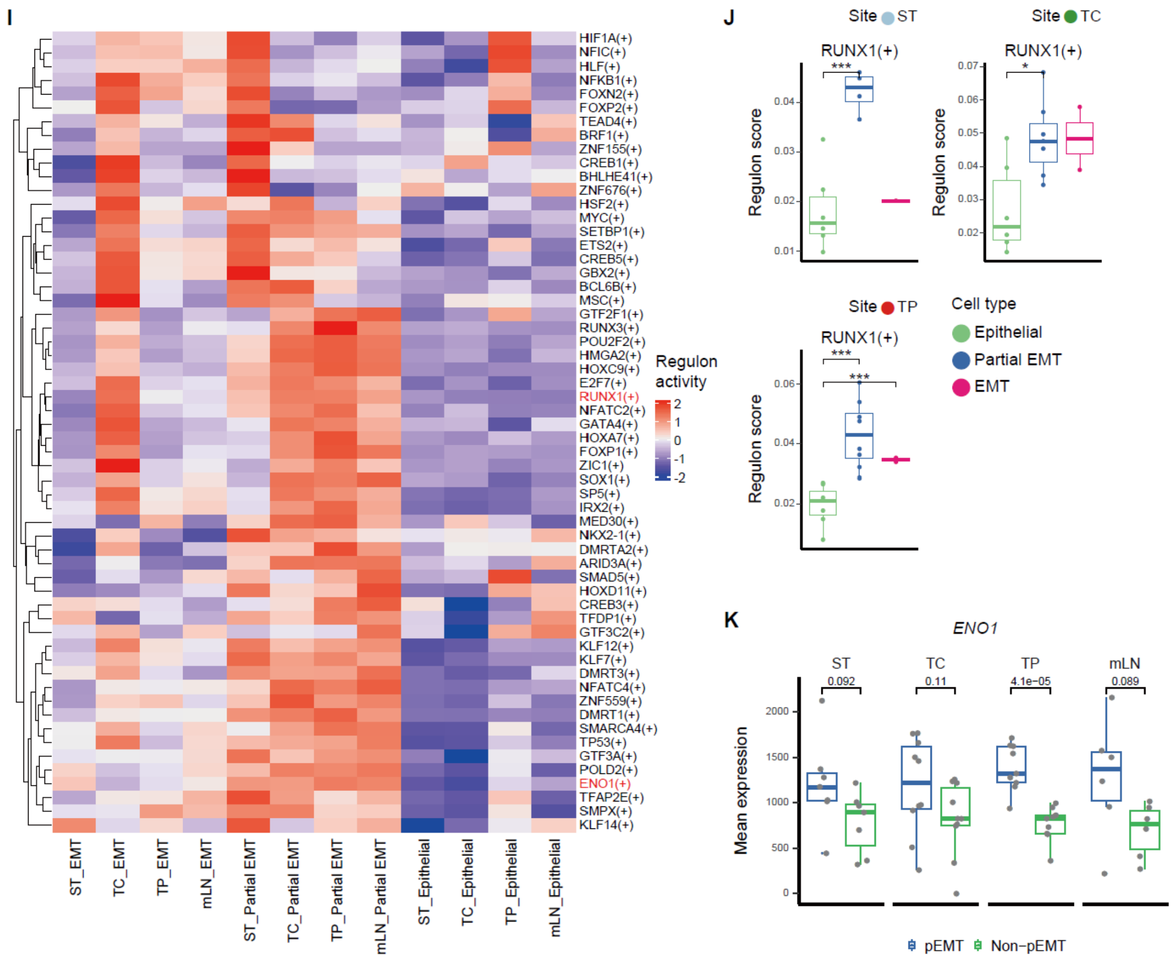
I
HIF1A(+)
NFIC(+)
HLF(+)
NFKB1(+)
FOXN2(+)
FOXP2(+)
TEAD4(+)
BRF1(+)
ZNF155(+)
CREB1(+)
BHLHE41(+)
ZNF676(+)
HSF2(+)
MYC(+)
SETBP1(+)
ETS2(+)
CREB5(+)
GBX2(+)
BCL6B(+)
MSC(+)
GTF2F1(+)
RUNX3(+)
POU2F2(+)
HMGA2(+)
HOXC9(+)
E2F7(+)
RUNX1(+)
NFATC2(+)
GATA4(+)
HOXA7(+)
FOXP1(+)
ZIC1(+)
SOX1(+)
SP5(+)
IRX2(+)
MED30(+)
NKX2-1(+)
DMRTA2(+)
ARID3A(+)
SMAD5(+)
HOXD11(+)
CREB3(+)
TFDP1(+)
GTF3C2(+)
KLF12(+)
KLF7(+)
DMRT3(+)
NFATC4(+)
ZNF559(+)
DMRT1(+)
SMARCA4(+)
TP53(+)
GTF3A(+)
POLD2(+)
ENO1(+)
TFAP2E(+)
SMPX(+)
KLF14(+)
Regulon activity
2
1
0
-1
-2
ST_EMT
TC_EMT
TP_EMT
mLN_EMT
ST_Partial EMT
TC_Partial EMT
TP_Partial EMT
mLN_Partial EMT
ST_Epithelial
TC_Epithelial
TP_Epithelial
mLN_Epithelial
J
Site ST
RUNX1(+)
***
Regulon score
0.04
0.03
0.02
0.01
Site TC
RUNX1(+)
*
Regulon score
0.07
0.06
0.05
0.04
0.03
0.02
Site TP
RUNX1(+)
***
***
Regulon score
0.06
0.04
0.02
Cell type
Epithelial
Partial EMT
EMT
K
ENO1
ST
TC
TP
mLN
0.092
0.11
4.1e-05
0.089
Mean expression
2000
1500
1000
500
0
pEMT
Non-pEMT

L

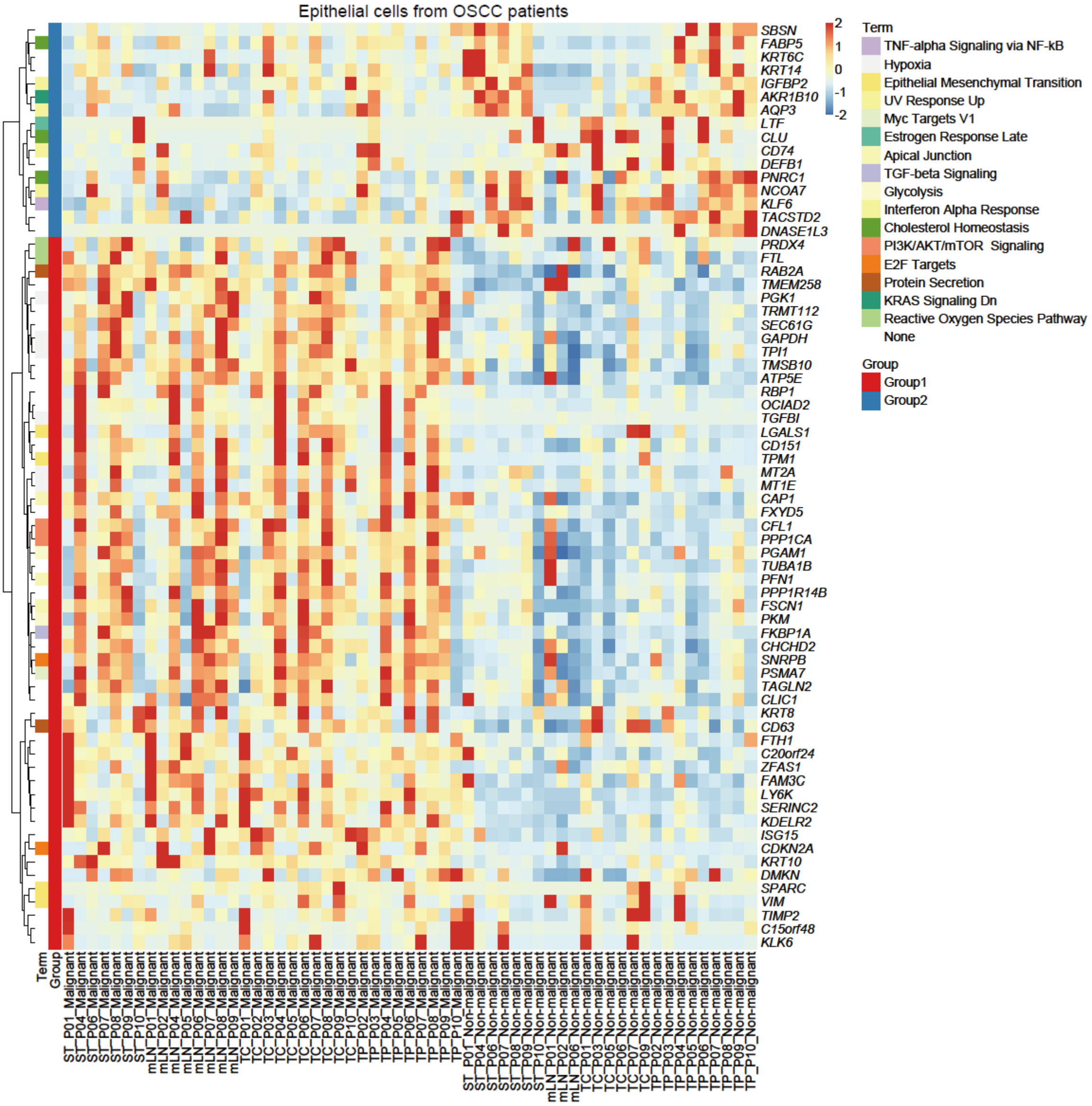
Epithelial cells from OSCC patients
SBSN
FABP5
KRT6C
KRT14
IGFBP2
AKR1B10
AQP3
LTF
CLU
CD74
DEFB1
PNRC1
NCOA7
KLF6
TACSTD2
DNASE1L3
PRDX4
FTL
RAB2A
TMEM258
PGK1
TRMT112
SEC61G
GAPDH
TPI1
TMSB10
ATP5E
RBP1
OCIAD2
TGFBI
LGALS1
CD151
TPM1
MT2A
MT1E
CAP1
FXYD5
CFL1
PPP1CA
PGAM1
TUBA1B
PFN1
PPP1R14B
FSCN1
PKM
FKBP1A
CHCHD2
SNRPB
PSMA7
TAGLN2
CLIC1
KRT8
CD63
FTH1
C20orf24
ZFAS1
FAM3C
LY6K
SERINC2
KDELR2
ISG15
CDKN2A
KRT10
DMKN
SPARC
VIM
TIMP2
C15orf48
KLK6
2
1
0
-1
-2
Term
TNF-alpha Signaling via NF-kB
Hypoxia
Epithelial Mesenchymal Transition
UV Response Up
Myc Targets V1
Estrogen Response Late
Apical Junction
TGF-beta Signaling
Glycolysis
Interferon Alpha Response
Cholesterol Homeostasis
PI3K/AKT/mTOR Signaling
E2F Targets
Protein Secretion
KRAS Signaling Dn
Reactive Oxygen Species Pathway
None
Group
Group1
Group2
Term
Group

M

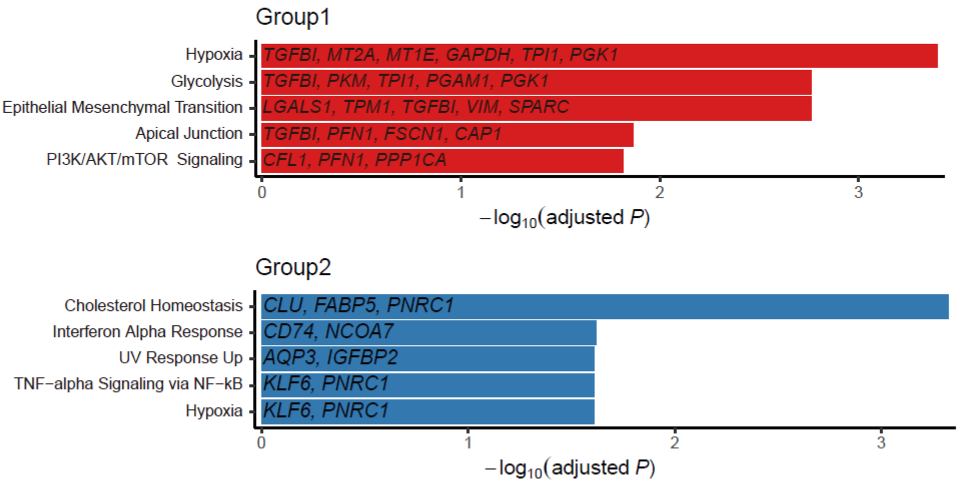
Group1
Hypoxia
Glycolysis
Epithelial Mesenchymal Transition
Apical Junction
PI3K/AKT/mTOR Signaling
TGFBI, MT2A, MT1E, GAPDH, TPI1, PGK1
TGFBI, PKM, TPI1, PGAM1, PGK1
LGALS1, TPM1, TGFBI, VIM, SPARC
TGFBI, PFN1, FSCN1, CAP1
CFL1, PFN1, PPP1CA
0
1
2
3
−log10(adjusted P)
Group2
Cholesterol Homeostasis
Interferon Alpha Response
UV Response Up
TNF−alpha Signaling via NF−kB
Hypoxia
CLU, FABP5, PNRC1
CD74, NCOA7
AQP3, IGFBP2
KLF6, PNRC1
KLF6, PNRC1
0
1
2
3
−log10(adjusted P)

Figure S4. Identification of epithelial, p-EMT, and EMT cells in advanced OSCC tumors.

(A) Kaplan-Meier plot showing the association between the proportion of p-EMT subtype, as inferred by BayesPrism, and overall survival in the TCGA HPV-negative HNSCC cohort. Patients were divided into high and low groups based on the median proportion of p-EMT cells.

(B) Left panel: Kaplan-Meier plot showing the association between the ratio of Epithelial subtype and overall survival in the TCGA HPV-negative HNSCC cohort. Right panel: Kaplan-Meier plot showing the association between the ratio of p-EMT to Epithelial subtypes and overall survival in the TCGA HPV-negative HNSCC cohort. The proportion of p-EMT and Epithelial subtypes was inferred by BayesPrism. The high and low groups are divided into high and low ratio groups based on the median ratio.

(C,D) Violin plots showing the expression levels of glycolysis-related genes (C) or hypoxia-related genes (D) in three epithelial subpopulations.

(E,F) Scatter plots of all epithelial cells from the published HNSCC scRNA-seq studies, showing correlation between the expression of p-EMT-related genes with glycolysis-related genes (E), and with hypoxia-related genes (F) at the single-cell level. Pearson's correlation (r) and associated p value are reported inside the scatter plot.

(G,H) Scatter plots of all HPV-negative HNSCC samples in the TCGA cohort, showing correlation between the mean expression of p-EMT-related genes with that of glycolysis-related genes (G), and with that of hypoxia-related genes (H). Pearson's correlation (r) and associated *p* value are reported inside the scatter plot.

(I) Heatmap depicting the regulon activity scores of p-EMT specific regulons, as identified by pySCENIC, across different tumor sites (ST, TC, TP, mLN) and subtypes (Epithelial, p-EMT, EMT).

(J) Box plots showing the distribution of RUNX1 regulon activity scores across different tumor sites (ST, TC, TP) and cell subtypes (Epithelial, p-EMT, EMT). Each dot represents

the mean RUNX1 regulon activity score of cells belonging to a specific subtype in each sample. Statistical significance between subtypes was determined using t-tests (*$p < 0.05$, **$p < 0.01$, ***$p < 0.001$).

(K) Box plots showing the distribution of mean *ENO1* expression in p-EMT and non-p-EMT cells across different tumor sites (ST, TC, TP, mLN). Statistical significance was determined using t-tests.

(L) Heatmap depicting the differentially expressed genes of malignant or non-malignant cells, as identified by inferCNV, across different tumor sites (ST, TC, TP, mLN) within patients. Genes were selected based on consistent expression patterns in at least three patients.

(M) Bar plots showing the top five significantly enriched pathways in malignant (Group 1) and non-malignant (Group 2) epithelial cells based on upregulated genes.

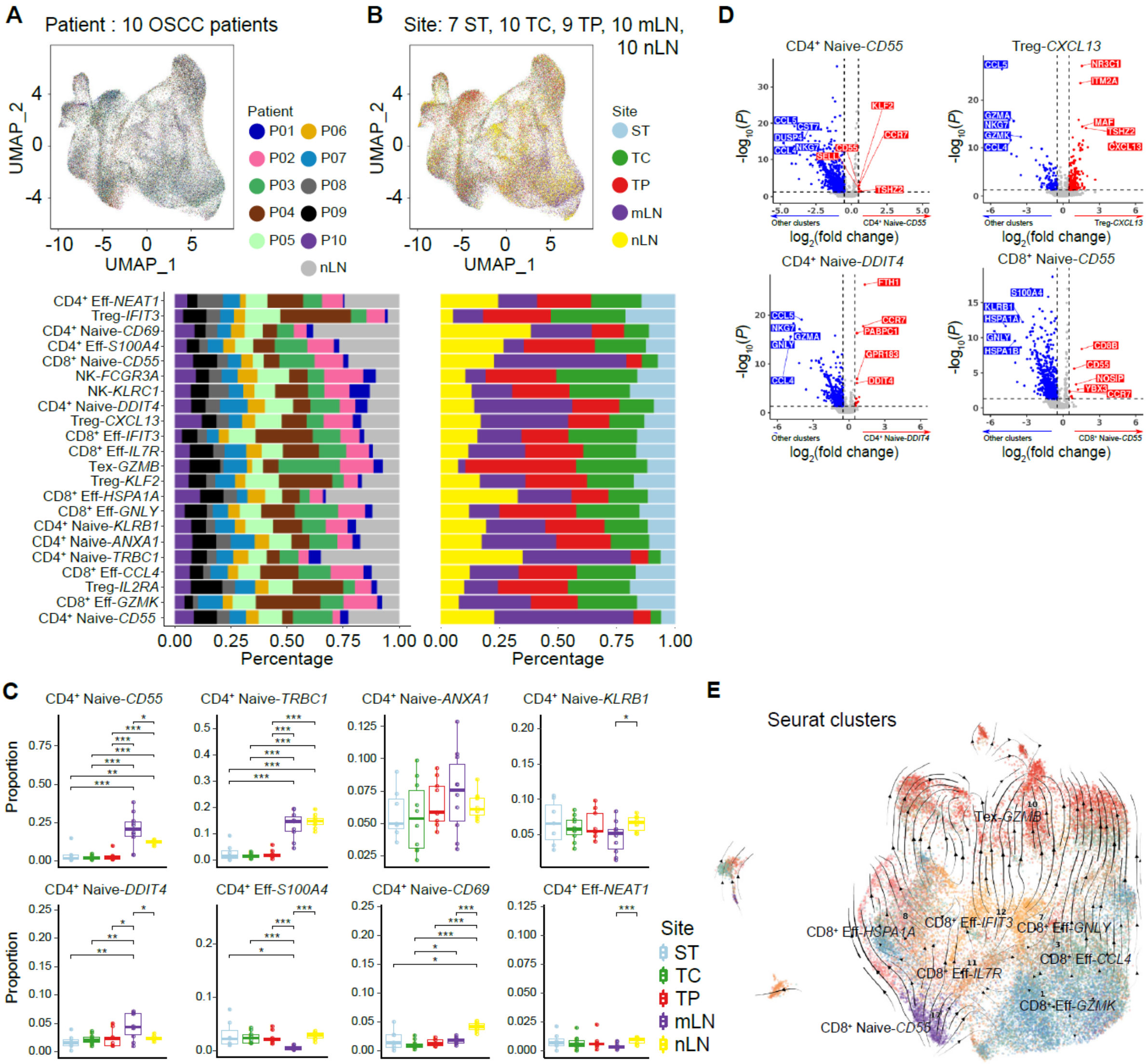
A
Patient : 10 OSCC patients
B
Site: 7 ST, 10 TC, 9 TP, 10 mLN, 10 nLN
D
CD4+ Naive-CD55
Treg-CXCL13
CD4+ Naive-DDIT4
CD8+ Naive-CD55
C
E
Seurat clusters
Site
ST
TC
TP
mLN
nLN
Percentage
Proportion
UMAP_1
UMAP_2

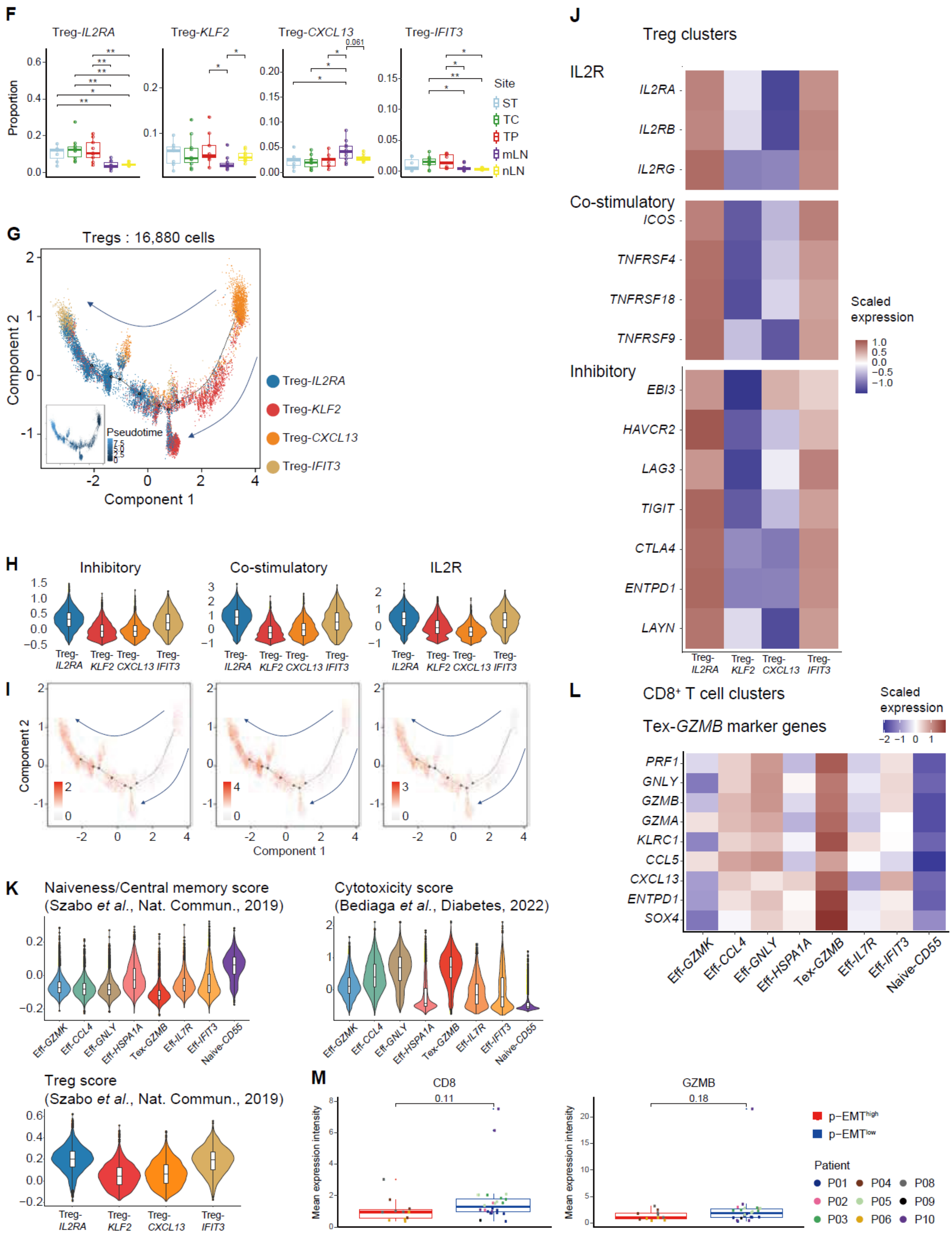

Figure S5. Diversity of T/NK cell populations across all lesions from advanced OSCC.

(A,B) UMAP and bar plots showing the proportions of patients (A) and sites (B) in each cluster in T/NK cells.

(C) Proportion distributions of $CD4^+$ naïve and $CD4^+$ effector T cell clusters across sampling sites.

(D) Volcano plots showing DEGs of $CD4^+$ naïve-*CD55*, Treg-*CXCL13*, naïve-*DDIT4*, and $CD8^+$ naïve-*CD55*. DEGs were identified using a pseudobulk approach.

(E) RNA velocity analysis of CD8 T cell lineages using scVelo. Each dot represents an individual cell, colored by the seurat clusters. Arrows indicate the inferred direction of cellular development, suggesting a lineage trajectory from CD8 naïve T cells to CD8 effector T cells and eventually to Tex cells.

(F) Proportion distributions of Treg clusters across sampling sites.

(G) The developmental trajectories of Treg cells by Monocle2 analysis. The arrows indicate differentiation pathways. Individual dots represent single cells, while different colors denote distinct Treg clusters. The arrows indicate differentiation pathways. The inlet plot showed cells colored by their corresponding pseudotime.

(H,I) Violin plots showing inhibitory, co-stimulatory, and IL2R scores for each Treg cluster (H), along with score changes across pseudotime (I).

(J) Heatmap showing the scaled normalized mean expression of Treg functional markers (rows) for each Treg cell cluster (columns).

(K) Violin plots showing T cell naiveness/central memory, cytotoxicity scores for each $CD8^+$ T cell cluster, and Treg score for each Treg cluster.

(L) Heatmap showing the scaled normalized mean expression of Tex-*GZMB* markers (rows) for each CD8 T cell cluster (columns).

(M) Box plots showing the mean expression intensity of CD8 and GZMB in p-EMT^{high} and p-EMT^{low} groups. Statistical significance was determined by two-sided t-test (*p < 0.05, **p < 0.01, ***p < 0.001).

Significance of differential proportion (*P* value) between sites was determined by two-sided t-test (box central lines, median; box limits, 25th and 75th percentiles; whiskers, 1.5x the interquartile range; * *p* < 0.05, ** *p* < 0.01, *** *p* < 0.001).

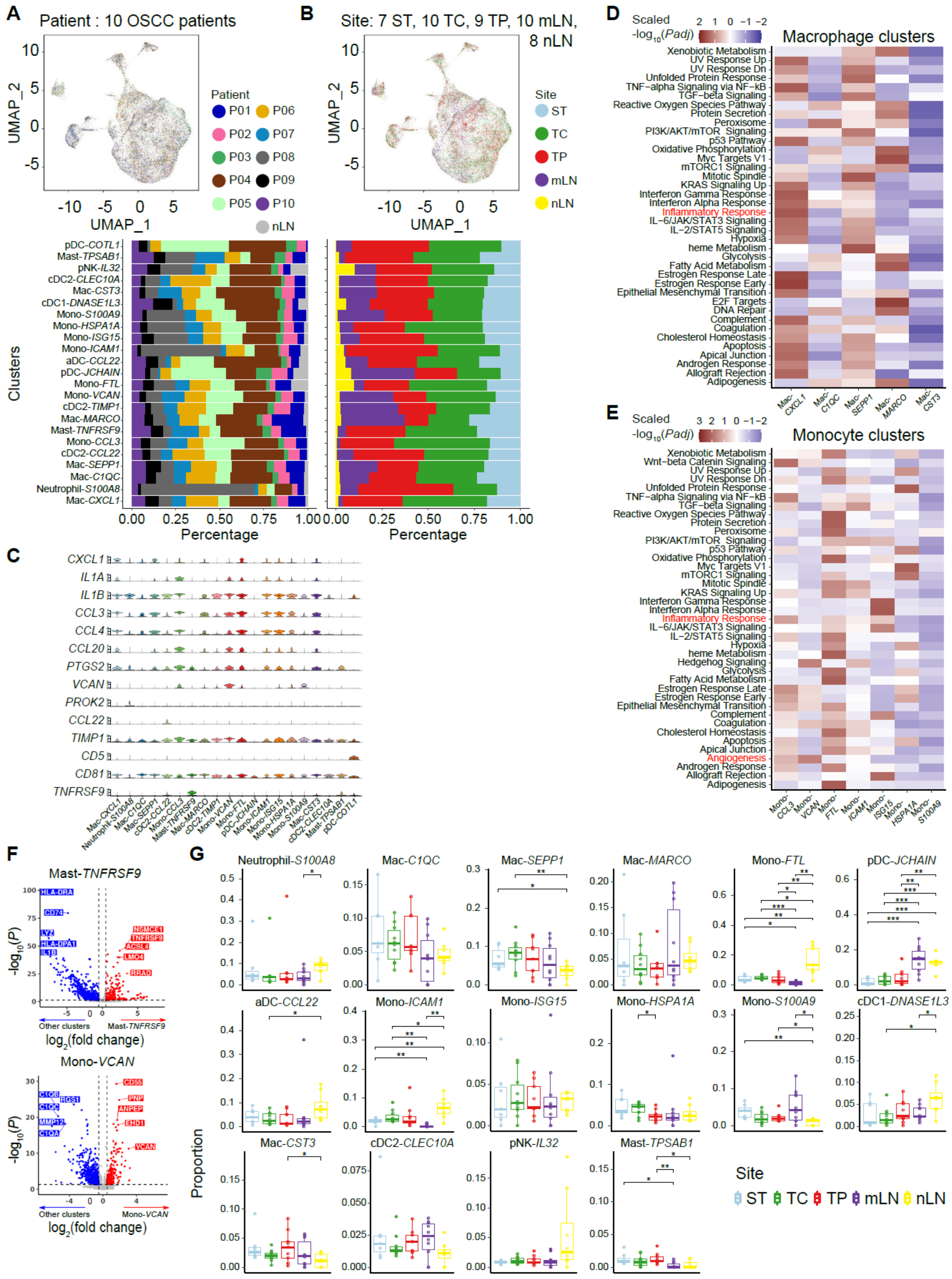
A
Patient : 10 OSCC patients
B
Site: 7 ST, 10 TC, 9 TP, 10 mLN, 8 nLN
UMAP_1
UMAP_2
Patient
P01 P02 P03 P04 P05 P06 P07 P08 P09 P10 nLN
Site
ST TC TP mLN nLN
Clusters
Percentage
C
D
Macrophage clusters
E
Monocyte clusters
Scaled -log10(Padj)
Inflammatory Response
Angiogenesis
F
Mast-TNFRSF9
Mono-VCAN
Other clusters
log2(fold change)
-log10(P)
G
Proportion
Site
ST TC TP mLN nLN

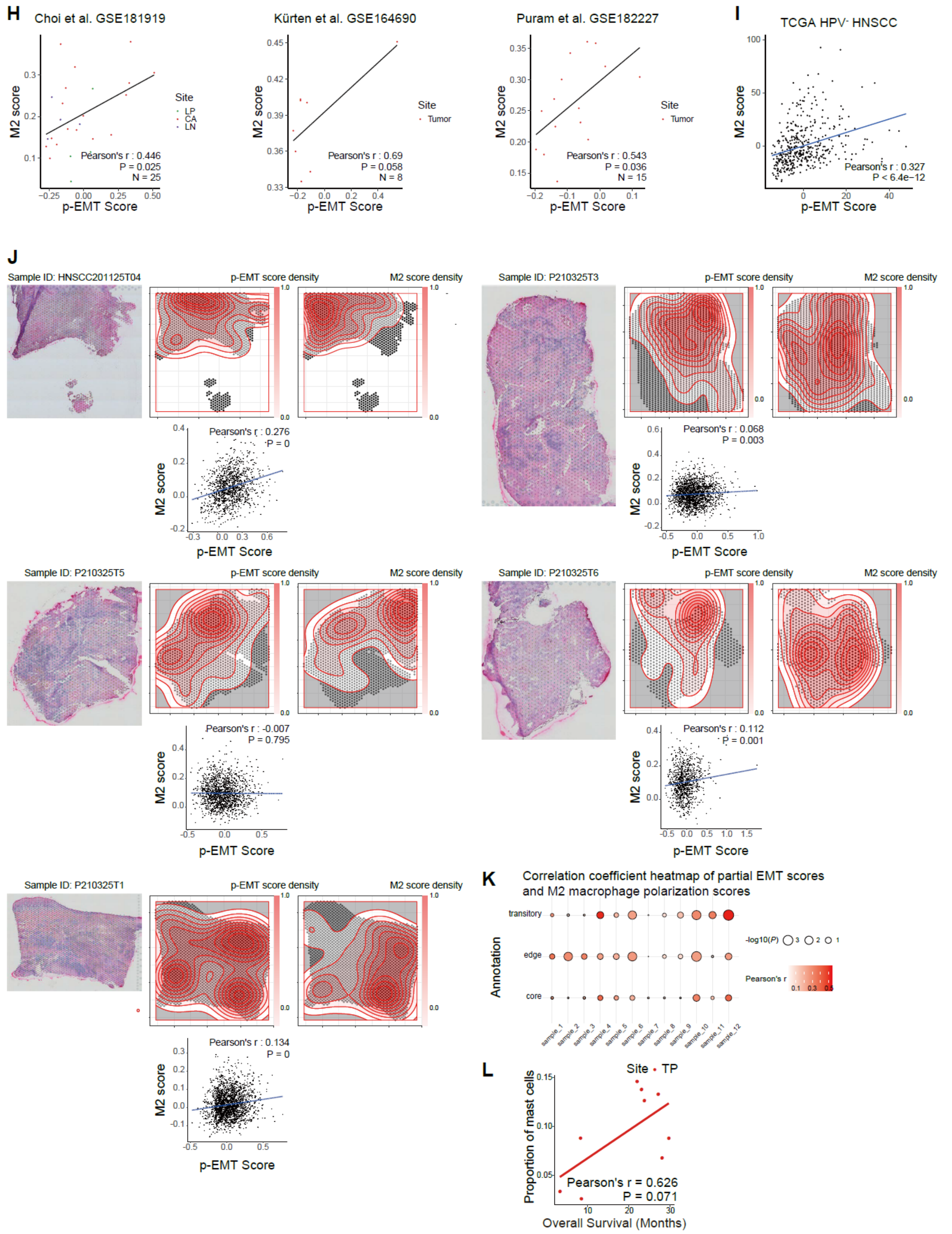

Figure S6. Diversity of myeloid cell populations across all lesions from advanced OSCC.

(A,B) UMAP and bar plots showing the proportions of patients (A) and sites (B) in each cluster in myeloid cells.

(C) Violin plots of the expression of genes highly expressed in the clusters with significant proportion differences across sampling sites. Only clusters of the same subtype were shown.

(D,E) Heatmap of the significantly enriched signaling pathways (rows) for each macrophage (D) or monocyte (E) cluster (columns).

(F) Volcano plots showing DEGs of C6:Mast-*TNFRSF9* and C9:Mono-*VCAN*. DEGs were identified using a pseudobulk approach.

(G) Proportion distributions of myeloid cell clusters not shown in Fig. 5C. Significance of differential proportion (P value) between sites was determined by t test (box central lines, median; box limits, 25th and 75th percentiles; whiskers, 1.5x the interquartile range; * $p < 0.05$, ** $p < 0.01$, *** $p < 0.001$).

(H) Scatter plots of p-EMT score versus M2 macrophage score in publicly available scRNA-seq datasets of HNSCC; LP: leukoplakia, CA: primary cancer, LN: metastatic tumors in the lymph nodes. Only samples with >= 25 macrophage cells and >= 25 epithelial cells were included. Pearson's correlation (r) and associated *p* value are reported inside the scatter plot.

(I) Scatter plot of all HPV-negative HNSCC samples in the TCGA cohort, showing correlation between the mean expression of p-EMT-related genes with that of M2 macrophage-related genes. Pearson's correlation (r) and associated *p* value are reported inside the scatter plot.

(J) Spatially mapped p-EMT and M2 macrophage scores in the publicly available Visium samples of stage IV OSCC patient. A correlation scatter plot between p-EMT and M2 scores for each spot is shown below.

(K) Correlation coefficient heatmaps for leading edge, transitory, and tumor core regions of each sample, visualizing the spatial heterogeneity in the correlation between p-EMT and M2 macrophage polarization.

(L) Scatter plot of OSCC TP samples, showing the positive correlation between the proportions of mast cells (consisting of Mast-*TNFRSF9* and Mast-*TPSAB1*) and overall survival. Pearson's correlation (r) and associated *p* value are reported inside the scatter plot.

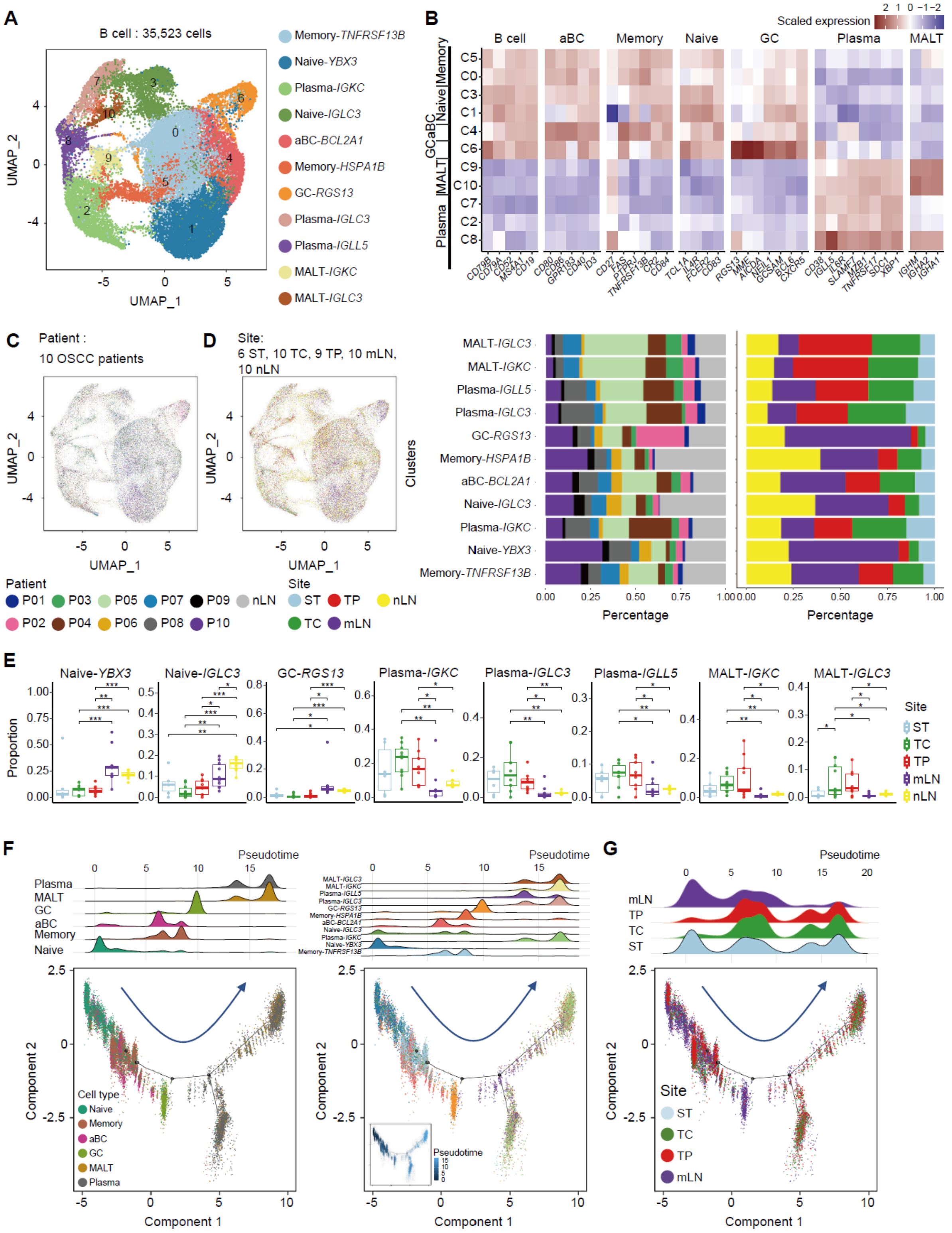

Figure S7. Diversity of B cell populations across all lesions from advanced OSCC.

(A) UMAP of B cells derived from all lesions, colored and labeled by cluster number, cell type and marker gene.

(B) Heatmap of scaled normalized expression of B cell marker genes.

(C,D) UMAP and bar plots showing the proportions of patients (C) and sites (D) in each cluster in B cells.

(E) Proportion distributions of eight representative B cell clusters with significant proportion differences across sampling sites. Significance of differential proportion (*P* value) between sites was determined by two-sided t-test (box central lines, median; box limits, 25th and 75th percentiles; whiskers, 1.5x the interquartile range; * $p < 0.05$, ** $p < 0.01$, *** $p < 0.001$).

(F) The developmental trajectories of B and plasma cells by Monocle2 analysis. Individual dots represent single cells, while different colors denote distinct cell subtypes (left) and clusters (right). The arrows indicate differentiation pathways. The inlet plot showed cells colored by their corresponding pseudotime.

(G) The developmental trajectories of B and plasma cells along the pseudotime. Each dot corresponds to a single cell, colored by site (right).

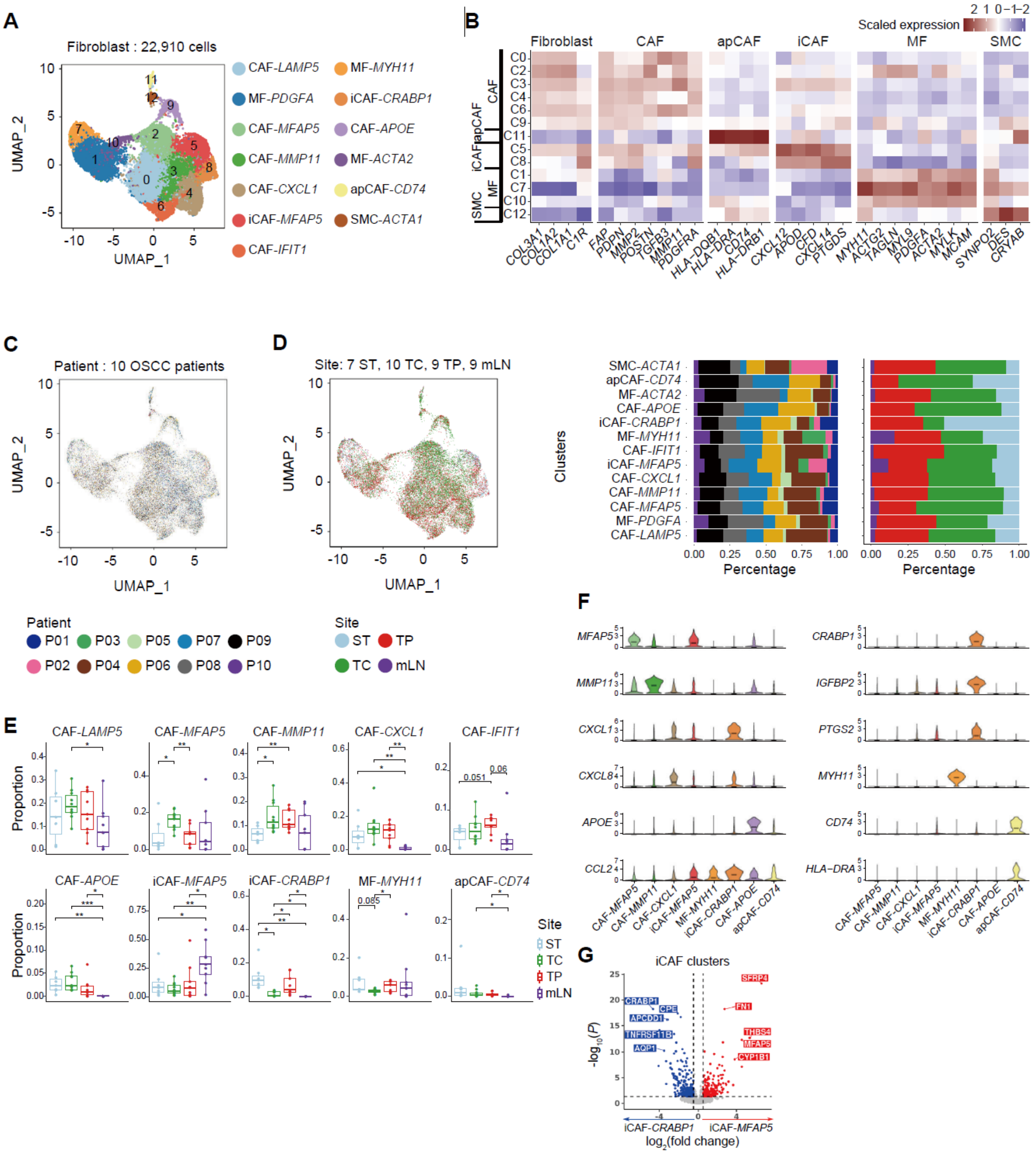

Figure S8. Diversity of fibroblasts across all lesions from advanced OSCC.

(A) UMAP of fibroblasts derived from all lesions, colored and labeled by cluster number, cell type and marker gene.

(B) Heatmap of scaled normalized expression of fibroblast marker genes.

(C,D) UMAP and bar plots showing the proportions of patients (C) and sites (D) in each cluster in fibroblasts.

(E) Proportion distributions of 10 representative fibroblast clusters with significant proportion

differences across sampling sites. Significance of differential proportion (*P* value) between sites was determined by two-sided t-test (box central lines, median; box limits, 25th and 75th percentiles; whiskers, 1.5x the interquartile range; * $p < 0.05$, ** $p < 0.01$, *** $p < 0.001$).

(F) Violin plots of the expression of highly expressed genes in fibroblast clusters with significant proportion differences across sampling sites. Only clusters of the same subtype were shown.

(G) Volcano plots showing DEGs of iCAF clusters. DEGs were identified using a pseudobulk approach.

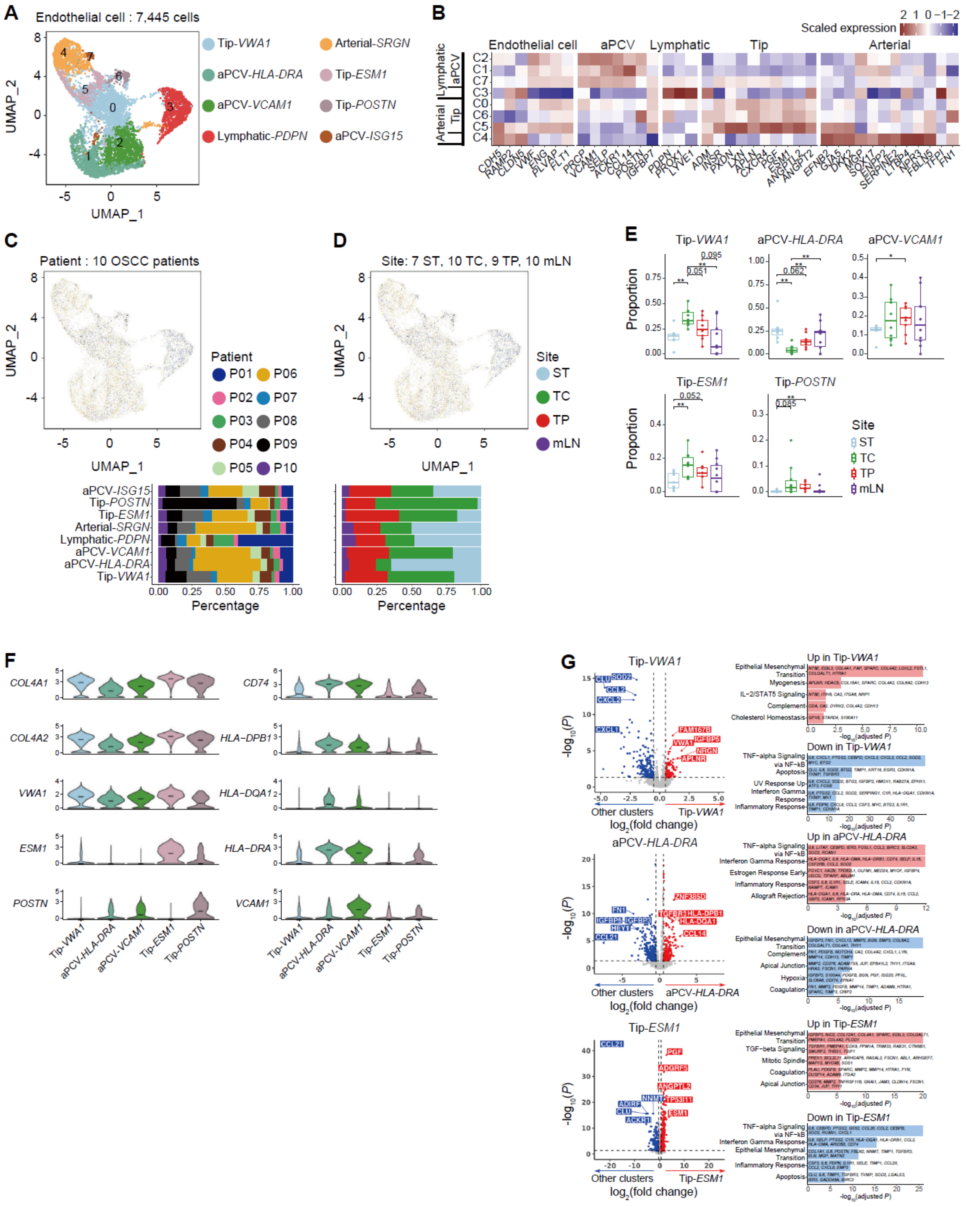

Figure S9. Diversity of endothelial cells across all lesions from advanced OSCC.

(A) UMAP of endothelial cells (ECs) derived from all lesions, colored and labeled by cluster number, cell type and marker gene.

(B) Heatmap of scaled normalized expression of EC marker genes.

(C,D) UMAP and bar plots showing the proportions of patients (C) and sites (D) in each cluster in endothelial cells.

(E) Proportion distributions of 5 representative EC clusters with significant proportion differences across sampling sites. Significance of differential proportion (*P* value) between sites was determined by two-sided t-test (box central lines, median; box limits, 25th and 75th percentiles; whiskers, 1.5x the interquartile range; * $p < 0.05$, ** $p < 0.01$, *** $p < 0.001$).

(F) Violin plots of the expression of highly expressed genes in EC clusters with significant proportion differences across sampling sites. Only clusters of the same subtype were shown.

(G) Volcano plots showing DEGs of Tip-*VWA1*, aPCV-*HLA-DRA*, and Tip-*ESM1*. DEGs were identified using a pseudobulk approach.

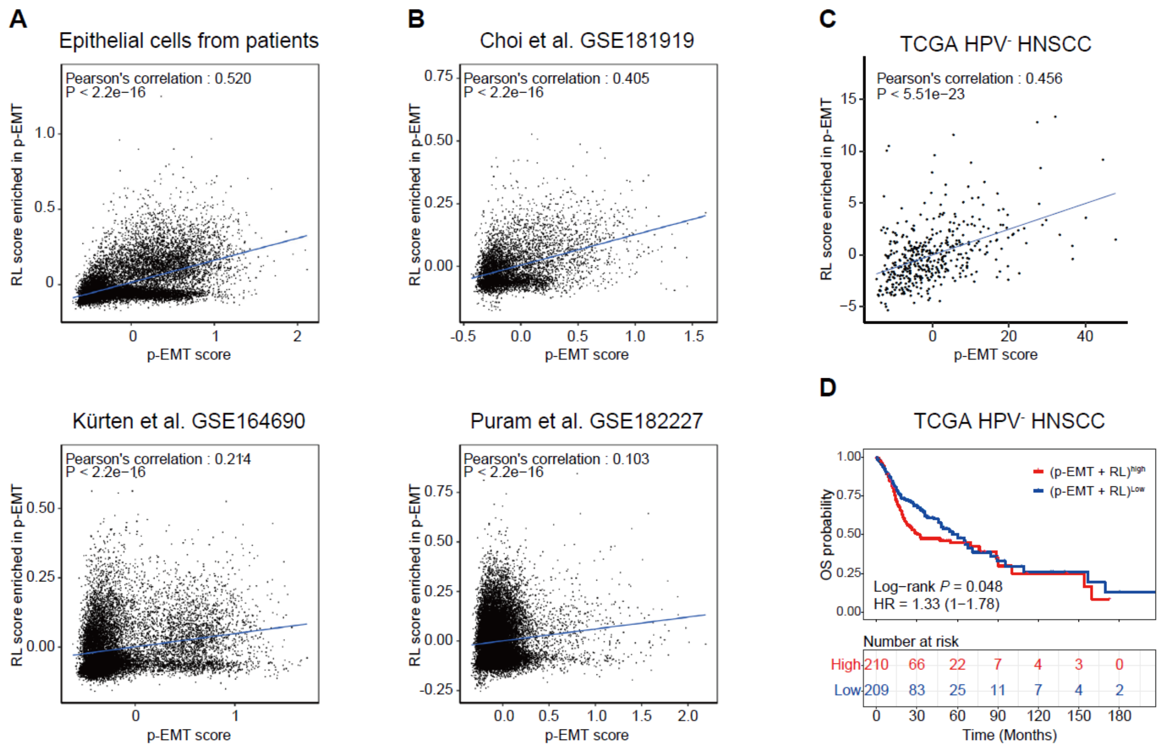

Figure S10. Score correlation of immunosuppressive receptors and ligands enriched in p-EMT cells with p-EMT markers.

(A,B) Scatter plots of all epithelial cells, showing correlation between the expression of p-EMT-related genes with that of receptors and ligands (RL) enriched in p-EMT cells (RL enriched in p-EMT) at the single-cell level both in our cohort (A) and in publicly available scRNA-seq datasets of HNSCC (B). Pearson's correlation (r) and associated *p* value are reported inside the scatter plot.

(C) Scatter plot of all HPV-negative HNSCC samples in the TCGA cohort, showing correlation between the mean expression of p-EMT-related genes with that of RL enriched in p-EMT. Pearson's correlation (r) and associated *p* value are reported inside the scatter plot.

(D) Kaplan-Meier plots showing that the patients with high expression of p-EMT and RL enriched in p-EMT markers (p-EMT + RL) have worse prognosis in the TCGA HPV-negative HNSCC cohort. The high and low groups are divided by the half value of the mean expression of the p-EMT + RL.
